# Tui: A Multigenerational and Expert-Correctable Tracker for Cellular Dynamics

**DOI:** 10.34133/csbj.0054

**Published:** 2026-04-24

**Authors:** I-Ming Chang, Hsieh-Fu Tsai

**Affiliations:** ^1^ Department of Biomedical Engineering, Chang Gung University, Taoyuan 333, Taiwan.; ^2^ Department of Neurosurgery, Chang Gung Memorial Hospital, Keelung, Keelung City 204, Taiwan.

## Abstract

Cell division, migration, and collective cell behaviors, along with less frequent events such as cell fusion, are fundamental biological processes underlying physiological and pathological mechanisms. However, accurate cell tracking and lineage reconstruction from time-lapse microscopy remain challenging because of failure modes such as identity switching and division misassignment, arising from dynamic cell behavior, morphological heterogeneity, and intermittent segmentation errors. We introduce Tui tracker, a multigenerational tracking framework that integrates integer linear programming optimization with lineage-aware corrections for both cell division and fusion. Although fully automated, the Tui tracker includes an expert-correctable graphical interface, allowing users to optionally inspect and refine lineage trees. We validate the Tui tracker using synthetic datasets and real-world data, including the DIC-C2DH-HeLa and Fluo-N2DH-SIM+ datasets from the Cell Tracking Challenge and T98G glioblastoma cell electrotaxis, achieving tracking accuracies of 0.983, 0.999, and 0.999, respectively. Beyond standard tracking accuracy metrics, the Tui tracker demonstrates superior performance on biologically motivated lineage fidelity measures such as BIO (averaging complete track reconstruction, branching correctness, track continuity, and cell cycle accuracy) and OP_CLB_ (integrating BIO with temporal association quality), thereby providing a more faithful reconstruction of cellular events. The framework accurately detects mitosis events and incorporates explicit modeling of fusion events, which are validated in controlled synthetic datasets due to the limited availability of annotated biological fusion data. By enabling robust, automated, and correctable analysis of complex cellular dynamics, the Tui tracker has broad applications in quantitative cell biology, cancer research, and high-content drug screening. The source code is available at https://github.com/hftsai/tui.

## Highlights

• Tui tracker utilizes an integer linear programming framework that jointly models cost for cell migration, mitosis, fusion, appearance, and disappearance, ensuring globally optimal lineage construction. Especially, fusion detection is capability absent in most existing state-of-the-art trackers.
• A graphical user interface that allows manual inspection and correction of lineage trees, integrating biological expertise with automation for reliable cell-lineage analysis.
• Broad applicability with annotation of the cell-tracking challenge format for lineage-association analysis and migration of 2D objects. Validated against synthetic data, the CTC DIC-C2DH-HeLa and Fluo-N2DH-SIM+ datasets, and the glioblastoma T98G electrotaxis dataset, achieving near-perfect tracking accuracy (TRA = 0.984, 0.999, and 0.999, respectively) and allowing high-content biological discovery.

## Graphical Abstract

**Figure:**
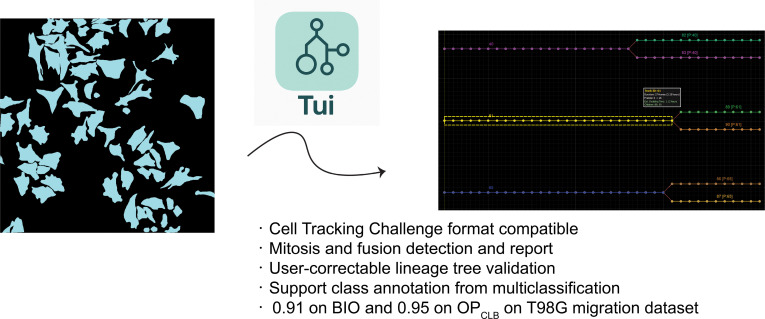

## Introduction

Cell division, fusion, migration, and collective cell behaviors are fundamental biological processes governing both physiological and pathophysiological mechanisms [1–3]. Lineage integrity, the reconstruction of parent–child relationships across generations, is central to understanding how these processes jointly shape tissue development, homeostasis, and disease. During embryonic development, tightly regulated migration ensures correct tissue and organ formation, while in adulthood, cell proliferation and migration together drive wound repair and tissue homeostasis [4,5]. Similarly, immune surveillance relies on rapid and dynamic behaviors of T cells, macrophages, and other immune effectors in response to infection, often involving transient receptor expression and cell–cell interactions [6,7]. Beyond individual cell behaviors, collective cell dynamics, where groups of cells coordinate movement and interactions, are fundamental to tissue morphogenesis, wound closure, and tumor invasion [8–10]. In microscopy-based analysis, spatially correlated motion and frequent neighbor exchanges in such systems challenge conventional tracking approaches, motivating the need for robust lineage-aware tracking frameworks.

Although single-cell multiomics techniques provide rich molecular characterization of cellular heterogeneity, they primarily capture static snapshots of cell states and rely on computational inference to reconstruct dynamic processes [11–13]. While such approaches have enabled insights into multiscale cellular interactions and population structure, they do not directly observe temporal lineage relationships or causal parent–child connections across cell generations. As a result, dynamic processes such as proliferation, differentiation, and cell–cell interactions are often inferred indirectly and may be confounded by noise, sampling limitations, and model assumptions [14,15].

In contrast, time-lapse microscopy provides direct temporal continuity, making quantitative single-cell tracking indispensable for resolving cellular behavior such as proliferation, migration, and cell–cell interactions [16,17]. Lineage continuity across cell generations enables direct observation of dynamic cellular processes at fine temporal resolution, which cannot be achieved by omics or positional tracking alone. In developmental biology and organoid research, lineage reconstruction reveals proliferation dynamics and differentiation hierarchies [18]. In oncology, lineage-resolved tracking helps characterize interactions between tumor clones and stromal or immune cells within the tumor microenvironment [19]. In biomanufacturing, it enables the detection of clonal drift and phenotypic instability that compromise reproducibility [20].

However, maintaining lineage integrity over long time sequences remains difficult. Identity switching (IDSW) and incorrect division assignment are common sources of lineage corruption [21,22]. Although cell fusion is a documented biological phenomenon in immune maturation, myogenesis, and cancer [23], most current tracking systems lack explicit mechanisms to detect it, typically misinterpreting fusion as disappearance, followed by spontaneous appearance [24].

The typical workflow to systematically understand the dynamic processes of cells by time-lapse microscopy involves image acquisition, segmentation, tracking, and lineage analysis. Advances in microscopy and imaging modalities have improved temporal resolution, but signal-to-noise issues and low spatial resolution still hinder segmentation accuracy [25–28]. Furthermore, traditional tracking strategies, such as nearest-neighbor assignment, linear assignment problem, and Kalman filtering, often fail under dense or highly motile conditions, leading to IDSW, fragmentation of lineage trees, and reduced robustness [4,29,30]. Manual tracking, although often prohibitively time-consuming, subjective, and unsuitable for high-throughput studies, is therefore still commonly used for lineage analysis [31,32]. Although previous efforts combining instance-aware deep-learning segmentation with particle-tracking algorithms have substantially improved automated analysis of cell dynamics [26,32,33], for example, in Usiigaci, cell migration in label-free phase contrast images can be tracked. However, many existing systems remain limited to simple one-to-one linking and cannot robustly resolve multigenerational lineage events such as mitosis or cell fusion.

Recent advances in machine learning have led to state-of-the-art (SOTA) methods such as Bayesian probabilistic tracking (e.g., Bayesian Tracker) [16], global optimization methods such as integer linear programming (ILP) and graph-based models [27,28,34], convolutional neural networks (CNNs) [35], and transformer-based trackers, such as Cell-TRACTR and TRACKASTRA [17,36]. These methods have improved accuracy in division detection and lineage reconstruction compared to conventional methods.

Despite these advances, critical challenges remain, particularly in maintaining lineage integrity under realistic imaging conditions. Intermittent segmentation errors, high cell density, and morphological variability frequently lead to IDSW, incorrect division assignment, and fragmentation of lineage trees [16,17,25,26].

Cell fusion detection presents an additional challenge, as 2 or more cells merge into a single entity [23]. Prior studies have noted that most tracking systems lack explicit mechanisms to model such events and instead represent them as cell disappearance, followed by new cell appearance, introducing systematic errors into reconstructed lineage trees [4,24,29].

Given the complexity of this task and the limitations of existing tools, the Cell Tracking Challenge (CTC) was established to provide standardized datasets, benchmarking protocols, and a collaborative platform for the community [4,5]. Importantly, evaluation metrics used in the CTC, including tracking accuracy (TRA) and biologically motivated lineage metrics, emphasize correct lineage reconstruction rather than simple positional TRA. Table 1 summarizes these capability gaps in representative tracking systems discussed above.

**Table 1.** Comparison of tracking system capabilities

Capabilities	TS [36]	UT [27]	TM [30]	BT [16]	Usiigaci [26]	Tui
Manual correction	×	✓	✓	×	✓	✓
Multialgorithm selection	✓	✓	✓	×	×	✓
Cell division detection	✓	✓	✓	✓	×	✓
CTC format output	✓	✓	✓	✓	×	✓
Lineage analysis	✓	✓	✓	✓	×	✓
Cell fusion detection	×	×	×	×	×	✓
CTC benchmark evaluation	×	✓	×	×	×	✓

CTC, Cell Tracking Challenge; TS, TRACKASTRA; UT, Ultrack; TM, TrackMate; BT, Bayesian Tracker (btrack); Tui, this work.

To tackle these challenges, we present the Tui tracker, a comprehensive framework that advances multigenerational lineage reconstruction in time-lapse microscopy with several innovations. The Tui tracker leverages the ILP optimization with the commercial high-performance Gurobi solver to jointly model cell division, fusion, and state transitions within a unified framework, enabling globally consistent solutions across the time series. By explicitly modeling division and fusion hypotheses, the framework supports improved parent–child assignment and reconstruction of lineage relationships. An intuitive graphical user interface (GUI) facilitates manual inspection, correction, and validation, making sophisticated algorithms accessible to biomedical researchers. In addition, model parameters can be calibrated using a Bayesian optimization strategy guided by macroscopic biological priors, such as expected cell counts and division frequencies. This enables experiment-specific adaptation without requiring ground-truth (GT) trajectory annotations.

## Methods

### Overview of the Tui cell-tracking framework

**Fig. 1.**
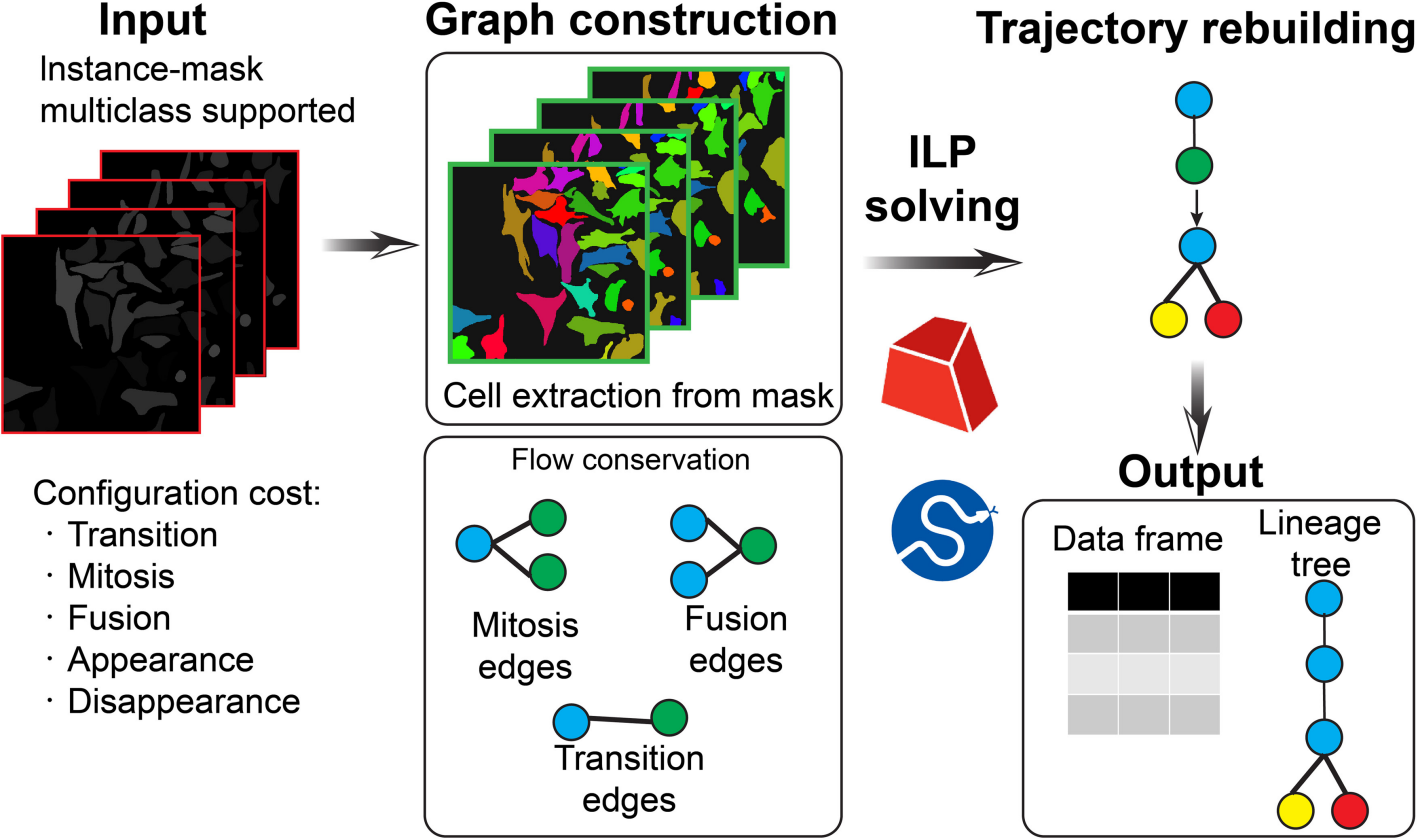
Integer linear programming (ILP)-based tracking pipeline. The pipeline processes sequential microscopy frames through a systematic workflow: cell detection from the instance masks, feature extraction, candidate association generation, constraint formulation incorporating biological priors, and global optimization of the costs between cell transition, mitosis, fusion, appearance, and disappearance to determine optimal cell trajectories and lineage relationships.

The proposed Tui-cell-tracking framework reconstructs multigenerational cell lineages from time-lapse microscopy data using an ILP-based optimization formulation (Fig. 1) in which the associations between the temporal frames of cells were determined by global optimization rather than local heuristics. The ILP formulation explicitly incorporated biological constraints and assigned quantitative costs to various cellular events, such as transition, appearance/disappearance, mitosis, and fusion, allowing the solver to identify globally optimal solutions. The resulting trajectories capture complex lineage relationships over time.

In addition to the proposed ILP tracking algorithm, the Tui tracker incorporates other tracking algorithms. First, a Trackpy-based simple cell-tracking algorithm previously reported in Usiigaci [26,37], which does not include mitosis detection capability, is included as a baseline tracking method (basic). Based on the Usiigaci algorithm, we further developed forward (FWD) and backward (BWD) tracking algorithms for identifying multigenerational mitosis events. In addition, we also integrated TRACKASTRA into the Tui tracker [36].

**Fig. 2.**
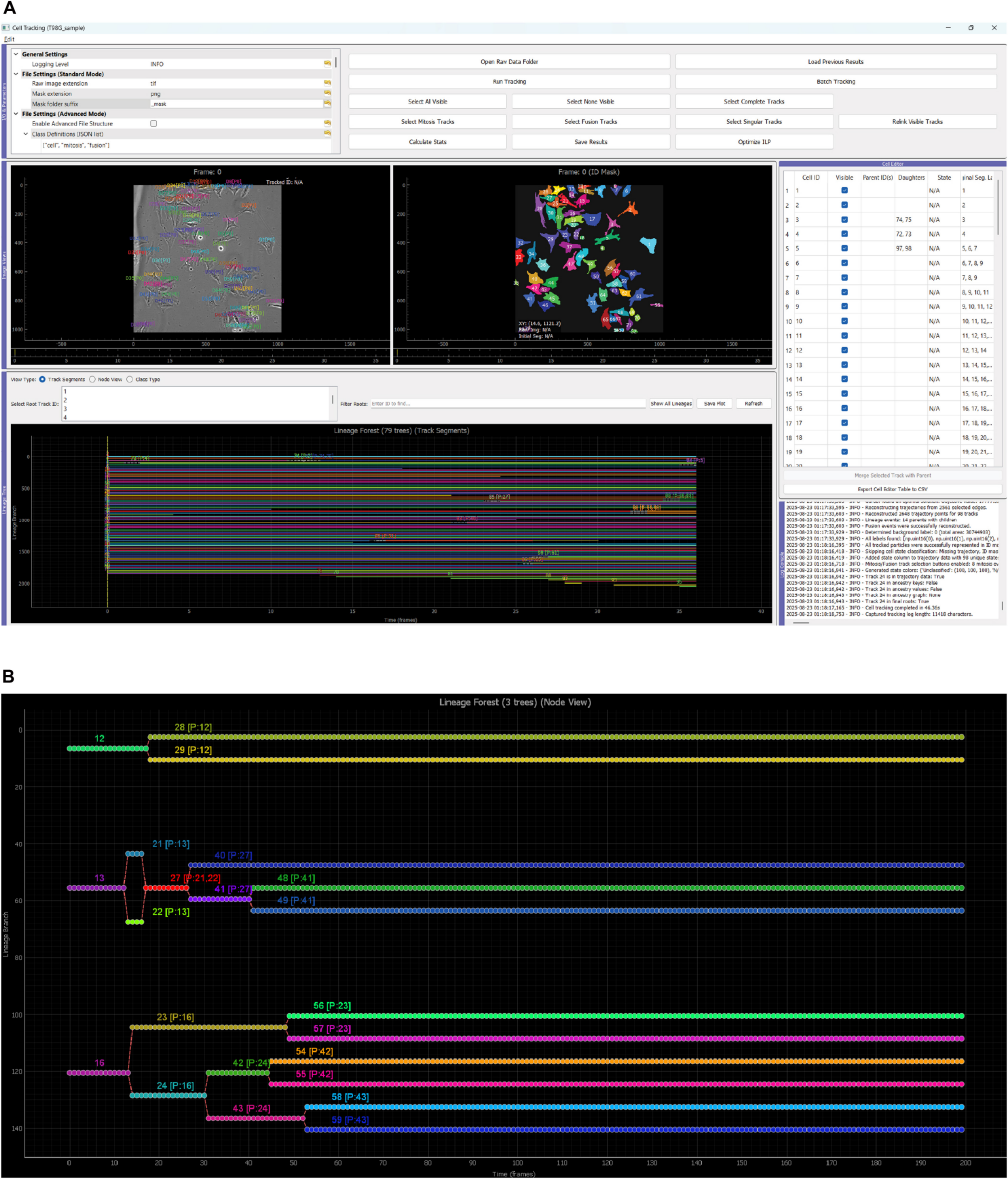
Tui tracker interface. (A) The graphical user interface enables the real-time visualization of raw microscopy images and masks of tracked cells and the lineage tree. The lineage tree can be visualized and edited in the editor table. (B) A display of the parent–child relationship of 3 tracks in a synthetic dataset. The node view enables users to manually correct and edit the lineage relationship. Note that the track in the middle undergoes multiple mitosis and fusion events captured by the Tui tracker correctly.

The Tui tracker includes a GUI (Fig. 2A) with an interactive-node-based lineage tree editor and a tabular editing interface. The GUI provides visualization of tracked image stacks, reconstructed lineage trees, and event annotations, serving as a central hub for reviewing and manually validating automated tracking results.

In the interactive-node-based lineage tree editor (Fig. 2B), users can select single or multiple trajectories for editing, manually adjust cell identities, merge or split tracks, and directly modify lineage links. Changes are immediately reflected in the lineage visualization, enabling seamless integration of manual corrections with automated tracking. Alternatively, parent–child relationships and cell classifications can be annotated in the editor table, which are then reflected in the lineage tree.

The mathematical framework of the Tui tracker addresses challenging scenarios encountered in live-cell imaging, including high cell density, complex multigenerational parent–child relationships, and rare cellular fusion events, which frequently confound conventional tracking algorithms [23]. In particular, as illustrated in Fig. 2B, the ILP-based method reconstructs complex multimitosis lineage trees and supports the identification and integration of fusion events without disrupting overall trajectory consistency, capabilities that are lacking in many existing solutions (Table 1).

### FWD and BWD Trackpy tracking algorithm for mitosis

The basic tracking algorithm in Trackpy was originally designed for particle tracking and does not natively support object splitting [26,37]. However, object splitting is common in live-cell imaging and represents key biological processes such as mitosis and cell differentiation.

We adopt a directional tracking strategy within the Trackpy framework in which newly appearing objects within a search radius are identified as potential mitotic events, following approaches reported previously [38,39].

The algorithm operates in both FWD and BWD modes to detect mitosis and fusion events and to reconstruct lineage relationships comprehensively (Fig. S1). A detailed description of the directional tracking algorithms is provided in Section S1.

#### Feature extraction and trajectory linking

For each frame, we extract quantitative morphological and physical features using scikit-image’s skimage.measure.regionprops [40], including centroid position $\left(x,y\right)$, area, equivalent diameter, perimeter, eccentricity, solidity, and orientation. Orientation is represented using $\sin\left(2\theta \right)$ and $\cos\left(2\theta \right)$ to provide rotation-invariant morphological descriptors.

Feature weighting emphasizes spatial and temporal consistency, with temporal weighting scaled according to the maximum displacement and memory gap parameters. All features are assembled into a pandas DataFrame and processed using Trackpy for initial trajectory linking, with configurable search range and memory parameters to handle temporary occlusions [37].

#### Event detection and lineage construction

The core module detects biological events based on tracking direction through systematic validation of candidate parent–child relationships. In FWD mode, for each parent candidate P in frame $F_{t}$, we search for daughter cells $D_{1}\;\text{and}\;D_{2}$ in frame $F_{t+1}$ to identify mitosis events. In BWD mode, for each potentially fused cell in frame $F_{t}$, we search for source cells in frame $F_{t-1}$ to detect fusion events.

Event validation uses 3 critical criteria, applied symmetrically for both mitosis and fusion detection:

• Distance constraint: $\begin{array}{c} ∥\mathrm{pos}\left(D_{i}\right)-\mathrm{pos}\left(P\right)∥<\alpha_{\text{dist}}\cdot \text{diameter}\left(P\right) \end{array}$
• Area conservation: $\beta_{\min}<\frac{A\left(D_{1}\right)+A\left(D_{2}\right)}{A\left(P\right)}<\beta_{\max}$
• Similarity: $\frac{\text{min}\left(A\left(D_{1}\right),A\left(D_{2}\right)\right)}{\text{max}\left(A\left(D_{1}\right),A\left(D_{2}\right)\right)}>\gamma_{\mathrm{sim}}$

where $\mathrm{pos}\left(\cdot \right)$ denotes the centroid position, $A\left(\cdot \right)$ denotes the cell area, and $\alpha_{\text{dist}}$, $\beta_{\min}$, $\beta_{\max}$, and $\gamma_{\mathrm{sim}}$ are configurable threshold parameters. These constraints enforce spatial proximity, approximate mass conservation, and morphological similarity between parent and daughter cells, ensuring biological plausibility in detected events.

The algorithm has a time complexity of $O\left(N\cdot C^{2}\right)$, where N denotes the total number of cell detections and C denotes the average number of cells per frame. The event detection step dominates computational cost due to exhaustive pairwise evaluation of potential parent–child relationships.

### ILP-based tracking algorithm and Bayesian optimization

To capture complex biological dynamics such as mitosis (1-to-2 splitting), fusion (2-to-1 merging), and the appearance or disappearance of cells, we formulate the tracking problem as an ILP on a spatiotemporal graph. The input is a 2-dimensional segmentation mask $M\in {\mathbb{Z}}^{H\times W\times T}$, where each frame $M_{t}$ contains labeled cell instances. Region-based analysis is used to extract individual cells, and each region $R_{i}^{t}$ is represented as a node $v_{i}^{t}$ with centroid, area, and morphological descriptors. Edges are then created between detections in adjacent frames to represent a standard transition, a mitotic split, or a fusion event.

Each edge e∈E is assigned a binary variable $x_{e}\in \left\{0,1\right\}$ and an event-specific cost $c_{e}$, defined as

$$c_{e}=\left\{\begin{array}{cc} w_{t}\cdot f_{\text{trans}}\left(v_{i}^{t},v_{j}^{t+1}\right), & \text{transition},\\ w_{m}+f_{\mathrm{mit}}\left(v_{i}^{t},v_{j}^{t+1},v_{k}^{t+1}\right), & \text{mitosis},\\ w_{f}+f_{\mathrm{fus}}\left(v_{i}^{t},v_{j}^{t},v_{k}^{t+1}\right), & \text{fusion}, \end{array}\right.$$ (1)

where the functions $f_{\text{trans}}$, $f_{\mathrm{mit}}$, and $f_{\mathrm{fus}}$ combine geometric displacement and relative size change to evaluate the plausibility of each hypothesized event. These functions favor small interframe motion and size stability for transitions, midpoint alignment and approximate area conservation for mitosis, and parent–child midpoint alignment with approximate area additivity for fusion, respectively. The global weights $w_{t}$, $w_{m}$, and $w_{f}$ regulate the relative penalty of transition, mitosis, and fusion events, thereby controlling the solver’s sensitivity to different lineage events. Explicit definitions and coefficient settings are provided in Table S3 and Section S2.

To incorporate biological constraints and account for objects entering or leaving the field of view (FOV), each node v∈V is associated with appearance and disappearance slack variables $a_{v},d_{v}\in \left\{0,1\right\}$, penalized by $w_{a}$ and $w_{d}$, respectively. The overall optimization minimizes the total cost of selected edges and boundary events:

$$\begin{array}{c} \underset{x,a,d}{\text{min}}\left(\sum_{e\in E}c_{e}x_{e}+w_{a}\sum_{v\in V}a_{v}+w_{d}\sum_{v\in V}d_{v}\right), \end{array}$$ (2)

subject to flow conservation constraints. These constraints enforce that, for every node, the sum of active incoming edges plus the appearance variable equals 1 and, similarly, the sum of outgoing edges plus the disappearance variable equals 1.

Rather than manually selecting the ILP cost parameters, we use a Bayesian optimization routine to calibrate the global weights (e.g., $w_{t}$, $w_{m}$, $w_{f}$, $w_{a}$, and $w_{d}$) and the linking distance $d_{\max}$. The optimizer repeatedly executes the ILP tracking pipeline using candidate parameter configurations and evaluates the resulting trajectories using a set of user-defined macroscopic biological targets. These targets include statistics observable by experimentalists, such as the number of cells in the first frame, the approximate number of mitosis events, the number of fusion events, and the total number of reconstructed tracks.

The objective function measures the normalized squared deviation between these predicted lineage statistics and the user-provided biological expectations. Because this optimization relies exclusively on experiment-level biological priors rather than GT trajectory annotations or benchmark metrics, it acts as an experiment-specific calibration step rather than a supervised training process, allowing the tracker to adapt to diverse datasets without introducing data leakage. The complete ILP algorithm is outlined below.

Having formulated the ILP tracker, the next step was to determine the cost weights and linking distance that control the balance between alternative lineage hypotheses. Instead of selecting these parameters manually, we used a Bayesian optimization framework implemented with gp_minimize from the scikit-optimize library.

In this procedure, the optimizer repeatedly runs the ILP tracking pipeline with different parameter settings. For each candidate parameter vector $\left(w_{t},\;w_{m},\;w_{f},\;w_{a},\;w_{d},\;\text{and}\;d_{\max}\right)$, a tracking graph is constructed, the ILP is solved (using either scipy.optimize.milp or Gurobi), and the resulting trajectories are reconstructed.

From these trajectories, several global lineage statistics are computed, including the number of cells in the first frame, the number of mitosis events, the number of fusion events, and the total number of reconstructed tracks. These statistics are compared with user-defined biological targets, and the discrepancy is measured using a normalized squared error. The optimizer then proposes new parameter settings and repeats the evaluation until the parameter configuration that best matches the specified biological targets is found.

Finally, we analyzed the computational complexity of the proposed ILP framework to assess its scalability. In the worst case, the runtime scales as $O\left(N\cdot C^{2}\right)$, where N denotes the total number of detections and C is the average number of cells per frame. This complexity arises from the enumeration of pairwise hypotheses required to model mitosis and fusion events, which introduce additional combinatorial possibilities compared to standard one-to-one linking. In practice, however, spatial pruning substantially reduces the number of candidate edges, resulting in near-linear runtime behavior for typical datasets. The space complexity is $O\left(N+E\right)$, where E represents the number of valid edges in the spatiotemporal graph. A full derivation of these bounds is provided in Section S2.


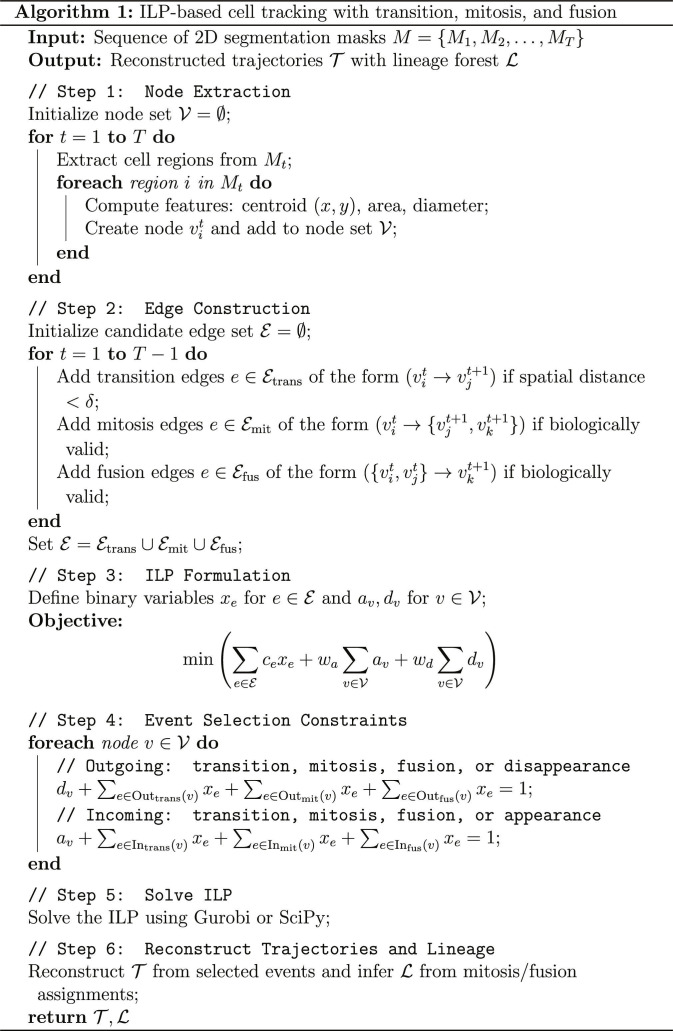


### Evaluation metrics

To rigorously assess the tracking performance of our proposed method, we used a comprehensive suite of evaluation metrics established by the CTC framework [41–44]. This standardized protocol allowed for systematic comparison with SOTA methods in the multifaceted assessment of object detection, segmentation, and tracking metrics.

The CTC metrics encompassed classical computer vision measures, such as segmentation accuracy (SEG), detection accuracy (DET), TRA, linking accuracy (LNK), multiple object TRA (MOTA) [45], and ID consistency (IDF1) [46]. These metrics collectively quantified detection quality, temporal association accuracy, and trajectory coherence.

In addition to these classical metrics, we emphasized biologically motivated indicators specifically designed for cell-tracking applications:

• HOTA [43]: Higher-order TRA is a unified metric that balances detection quality and temporal association accuracy.
• CHOTA [44]: Cell-specific higher-order TRA is an extension of HOTA that incorporates mitotic events and lineage structure, providing lineage-aware tracking assessment.
• CT [42]: Complete tracks measures the proportion of GT cell trajectories that are fully and correctly reconstructed without ID switches or fragmentation.
• BC [42]: Branching correctedness evaluates the accuracy of mitosis detection using an F1-based score on predicted and GT branching events.
• LNK: Linking accuracy evaluates how precisely each object is tracked in consecutive frames by comparing the acyclic directed graph produced by a tested algorithm with the reference graph provided by the GT [42].
• BIO: The biological accuracy measure averages biologically inspired measures applicable to a given dataset, including complete track reconstruction (CT), branching correctness (BC), track fraction (TF), and cell cycle accuracy (CCA), reflecting the fidelity of biological event reconstruction.

$$\mathrm{BIO}=0.25\times \left(\mathrm{CT}+\mathrm{BC}+\mathrm{TF}+\mathrm{CCA}\right)$$ (3)

• OP_CLB_: An overall linking performance score that integrated BIO and LNK, capturing both biological accuracy and temporal association quality.

$$OP_{\mathrm{CLB}}=0.5\times \left(\mathrm{LNK}+\mathrm{BIO}\right)$$ (4)

In our experiments, we specifically reported the BIO and OP_CLB_ metrics, which emphasize biologically meaningful linkage accuracy. All metric calculations were performed using the py-ctcmetrics library [5,44], which strictly adhered to the CTC evaluation protocol to ensure reproducibility and fair comparison. Detailed mathematical definitions and computation steps for each metric are provided in Section S3.

### Datasets and experimental setup

To comprehensively evaluate our tracking framework, we used synthetic and real-world datasets that represent different challenges and validation scenarios in cell-tracking research. A Python script included in the source code was developed in-house to generate synthetic datasets in which users can configure the number of cells undergoing mitosis and fusion throughout a time sequence. In addition to synthetic images and segmentation masks, a GT lineage graph compliant with the CTC format was generated for benchmarking.

The synthetic dataset was created to simulate realistic cell behaviors, including migration, division, and fusion events within a 200-frame time-lapse sequence. The synthetic environment modeled a population of cells undergoing stochastic migration with biologically plausible velocity constraints. Specifically, cell motion was modeled as a bounded random walk process with a fixed base velocity and Gaussian perturbations, while mitosis timing was sampled from configurable lifespan intervals and fusion events were triggered on the basis of spatial proximity thresholds. The dataset contained 41 mitosis events and 1 fusion event distributed throughout the sequence. Cell morphologies and movements were designed to mimic real biological behavior while maintaining computational tractability for exhaustive GT annotation.

To further evaluate the capability of the proposed framework to handle complex lineage topologies involving both mitosis and fusion events, we also generated an additional synthetic sequence containing 6 explicitly defined fusion events. This controlled dataset allowed rigorous validation of the algorithm’s ability to resolve both 1-to-2 division and 2-to-1 merging relationships in lineage trees.

For benchmarking tracker performance, real-world datasets must be accompanied by high-quality GT annotations. First, we chose a high-difficulty differential interference contrast (DIC) microscopy dataset (DIC-C2DH-HeLa) from the CTC [41], which includes a gold-standard GT lineage annotation. We also trained a CNN-based segmentation model using Detectron2 [47,48] to generate instance masks for the HeLa dataset.

In the DIC-C2DH-HeLa dataset used in this study, the number of cells per field of view (FOV) ranges from 12 in the first frame to 21 in the last frame as the population proliferates. Each sequence contains 83 frames and includes 9 mitosis events, with lineage trees extending up to 2 generations at an interval of 10 min. Confluency increases from 39.6% in the first frame to 62.2% in later frames, as estimated by converting instance masks to binary masks and measuring the occupied area using ImageJ.

Second, to evaluate tracking performance under directional migration conditions, we used an internal dataset consisting of 37 frames capturing 6 h of electrotaxis in a glioblastoma cell line (T98G) under a direct current electric field of 3 V/m, as previously reported [48]. In this dataset, segmentation masks were manually traced at whole-cell pixel resolution and curated to produce a gold-standard GT lineage annotation.

The T98G dataset contains 70 cells in the first frame and 72 cells in the final frame. The imaging sequence includes 37 frames and 8 mitosis events, with lineage trees extending up to 2 generations. Cell confluency decreases from 24.9% to 18.8% during the experiment as cells migrate directionally under the applied electric field. Cell migration speeds in this dataset are on the order of 5 to 15 μm/h and exhibit a directional bias consistent with electrotactic response [48].

In addition, we evaluated the framework on the Fluo-N2DH-SIM+ dataset from the CTC, which consists of 65 frames with cell counts increasing from 30 in the first frame to 41 in the final frame. Due to cells entering and leaving the FOV, the sequence exhibits dynamic population changes. The dataset includes 29 mitosis events, with lineage trees extending up to 3 generations, and is acquired at a temporal resolution of 29 min per frame.

We note that unambiguously annotated biological fusion events are not present in these real datasets, which primarily represent standard proliferation and migration assays. Fusion events are known to occur in specialized biological contexts such as immune cell fusion, myoblast fusion during muscle development, syncytium formation, and engineered cell fusion systems in tissue engineering [23]. Because publicly available time-lapse datasets containing validated fusion annotations are extremely rare, fusion-tracking capabilities were validated using controlled synthetic datasets where the GT topology is explicitly defined.

To improve reproducibility and accessibility, example datasets used in this study, including synthetic datasets, segmentation masks, curated lineage annotations, and example tracking results, are publicly available on Zenodo at https://zenodo.org/records/19026908.

All datasets were benchmarked and compared using multiple tracking methods, including a basic tracking method (Trackpy) [26], FWD/BWD directional tracking (this work), the ILP-based method (this work), a transformer-based state-of-the-art tracker (TRACKASTRA) [36], and TrackMate (a widely used ImageJ plugin) [30], when available. To ensure that the Trackpy-based tracker produced tracking data fully compatible with CTC evaluation metrics, we configured the algorithm with memory = 0.

The experiments were carried out on a Linux workstation equipped with an Intel i7-13700 processor and an NVIDIA Quadro RTX 6000 graphics card. When the ILP method was used to optimize trajectories, the Gurobi solver [49] was used unless otherwise noted.

### Parameter sensitivity analysis

To characterize the robustness of the ILP tracking framework to parameter selection, we performed a systematic grid search over the mitosis cost ($w_{m}$) and fusion cost ($w_{f}$) parameter space. The search spanned $w_{m}\in \left[1,20\right]$ and $w_{f}\in \left[0,25\right]$ at various intervals, yielding 15 experimental conditions. All other ILP parameters were held constant at default values: transition cost weight $w_{t}=1$, appearance cost $w_{a}=20$, disappearance cost $w_{d}=20$, and maximum link distance $d_{\max}=50$ pixels (Table S4). For each parameter combination, tracking was performed on the complete image sequence and the TRA, CHOTA, BIO, and OP_CLB_ metrics were computed against GT annotations following the CTC evaluation protocol. The resulting accuracy values were interpolated onto a regular grid and visualized as topological phase diagrams to identify distinct performance regimes and optimal operating regions. This analysis was conducted across 4 experimental conditions: HeLa and T98G datasets with either GT or Detectron2-generated segmentation masks, enabling the assessment of how segmentation quality influences parameter sensitivity.

## Results

### Evaluation on computer-generated synthetic dataset

We first benchmarked the performance of trackers using a synthetic dataset in which a perfect GT mask set and GT CTC-compliant format could be automatically generated.

As shown in Table 2, both our ILP-based method and TRACKASTRA configurations demonstrated performance close to the GT. Specifically, ILP successfully reconstructed 99 unique tracks, which exactly matched the GT, while TRACKASTRA generated 106 tracks, slightly exceeding the GT. Automated tracking algorithms often produce an excessive number of trajectories relative to the true cell population, primarily due to trajectory fragmentation and spurious linking [50]. Such overestimation inflates track counts, distorts downstream quantitative analyses, and ultimately lowers confidence in the biological validity of the results. BWD tracking particularly suffers from fragmentation (483 tracks), while basic tracking fails to identify branching events, treating child cell tracks as their parents and thereby causing false-negative track identification.

**Table 2.** Tracking statistics of a synthetic dataset (200 frames). Best performance values are shown in bold.

Tracking statistics	GT	Basic	FWD	BWD	ILP	TS
Time (s)	NA	23.32	112.93	1,162.71	141.26	**29.12**
Total unique tracks	99	37	91	483	**99**	106
Average track length (frames)	95.11	165.19	103.47	19.49	**95.11**	88.83
Median track length (frames)	124	167	132	1	**124**	85.5
Mitosis events	41	0	23	221	**41**	43
Fusion events	1	0	0	0	**1**	0
Life cycle time min (frames)	4	4	1	1	**4**	1
Life cycle time max (frames)	200	200	200	19.49	200	200
Life cycle time average (frames)	95.11	165.19	103.47	19.49	**95.11**	88.83

GT, ground truth; FWD, forward; BWD, backward; ILP, this work; TS, TRACKASTRA.

In terms of the identification of biological events, our ILP-based method and TRACKASTRA detected 41 and 43 mitosis events, respectively, compared to the GT of 41 mitosis events, indicating robust division detection capabilities. Only our ILP-based method successfully detected the programmed fusion event in the synthetic benchmark, demonstrating the ability to capture merging topologies that are relevant in specialized biological contexts such as tissue engineering and immunology [6].

To further validate fusion-aware lineage reconstruction beyond the single fusion event in the 200-frame synthetic sequence, we generated an additional synthetic benchmark specifically designed to stress-test merging topologies. This new synthetic dataset contains 200 frames, 98 unique tracks, 34 mitosis events, and 6 explicitly defined fusion events. Using the known GT lineage topology, the ILP-based framework successfully reconstructed the complete lineage structure without track fragmentation, correctly resolving both 1-to-2 mitosis and 2-to-1 fusion events. Because the topology is explicitly defined, this benchmark eliminates ambiguity caused by temporary overlap or segmentation artifacts and provides direct validation of the fusion-tracking capability of Tui.

For cell life cycle accuracy, defined as the metric quantifying the cell population whose entire life cycle (mitosis to mitosis) is observed, our ILP-based method closely matched the GT in the average, minimum, and maximum life cycle durations, with an average life cycle time of 95.11 frames. TRACKASTRA achieved similar results but exhibited a slightly shorter average track length of 88.83 frames.

The CTC evaluation metrics for the synthetic dataset are summarized in Table 3. ILP achieved near-perfect TRA, while TRACKASTRA exhibited comparable performance, achieving similar TRA and MOTA scores and slightly outperforming our ILP-based method in metrics such as IDF1 (0.935), CHOTA (0.936), and TF (0.964), highlighting its strength in maintaining temporal consistency over long sequences.

**Table 3.** CTC metrics of a synthetic dataset (200 frames). Best performance values are shown in bold. ILP cost: 0.5, 1, 10, and 20 (transition, mitosis, fusion, and appearance/disappearance).

Metrics	GT	Basic	FWD	BWD	ILP	TS	TM
TRA	1	0.998	0.998	NA	**1**	**1**	0.950
SEG	1	1	1	NA	**1**	**1**	0.871
DET	1	1	1	NA	**1**	**1**	0.966
IDF1	1	0.824	0.844	NA	0.926	**0.935**	0.722
MOTA	1	0.996	0.994	NA	**0.998**	**0.998**	0.912
HOTA	1	0.847	0.865	NA	0.938	**0.952**	0.756
CHOTA	1	0.825	0.836	NA	0.921	**0.936**	0.726
LNK	1	0.987	0.985	NA	**0.997**	0.996	0.848
CT	1	0.256	0.253	NA	**0.849**	0.732	0
TF	1	0.863	0.892	NA	0.957	**0.964**	0.694
CCA	1	0	0	NA	**0.939**	0.874	0
MT	1	0.838	0.798	0.566	**0.899**	**0.899**	0.541
ML	0	0	0	0	0	0	0
IDSW	0	35	59	NA	**17**	18	187
TP	9,416	9,416	9,416	NA	9,416	9,416	9,407
FN	0	0	0	NA	0	0	9
FP	0	0	0	NA	0	0	0
Precision	1	1	1	NA	1	1	1
Recall	1	1	1	NA	1	1	0.999
BIO	1	0.280	0.364	NA	**0.930**	0.851	0.174
OP_CLB_	1	0.633	0.675	NA	**0.964**	0.924	0.511

BWD, backward; CCA, cell cycle accuracy; CT, complete track reconstruction; FN, false negative; FP, false positive; FWD, forward; GT, ground truth; IDSW, ID switching; ILP, this work; MT, mostly tracker; ML, mostly loss; NA, not applicable; TF, track fraction; TM, TrackMate; TP, true positive; TS, TRACKASTRA.

From a biological relevance perspective, our ILP-based method achieved the highest BIO and OP_CLB_ scores of 0.930 and 0.964, respectively, surpassing TRACKASTRA and all other methods. This indicates that ILP provides superior lineage reconstruction capabilities, which are crucial for accurately capturing cell divisions and parent–child relationships. In contrast, basic and FWD configurations, as well as TrackMate, lag behind in ID-aware metrics such as IDF1 and BIO, despite showing relatively high detection scores. The marked increase in ID switching (IDSW) in these methods contributes to an increase in the number of unique tracks, which can lower confidence in the interpretation of biological data. These results clearly indicate that simpler tracking approaches are less effective in handling dynamic cellular events such as mitosis and fusion and may yield misleading results that confound interpretation. Due to the long time sequence and the large number of cells, the problem complexity for BWD configuration tracking is too high for CTC metrics to be computed.

### CTC challenge: DIC-C2DH-HeLa and Fluo-N2DH-SIM+ benchmark datasets

To evaluate the performance of the tracking algorithm on real-world datasets, we analyzed 2 datasets from the CTC: the DIC-C2DH-HeLa dataset and the Fluo-N2DH-SIM+ dataset. These datasets represent complementary experimental conditions, enabling the evaluation of the tracker under both label-free imaging and fluorescence microscopy scenarios.

Following the official CTC protocol, the evaluation for the DIC-C2DH-HeLa dataset was performed using the training sequence (sequence 01), where the consortium provides manually curated GT annotations (01_GT), including segmentation masks and the lineage reference file (man_track.txt). The tracking metrics were computed using the official CTC evaluation tool (py-ctcmetrics), where the TRA score evaluates the consistency of the reconstructed lineage graph against the reference lineage and the SEG score compares segmentation masks on the available annotated frames.

For experiments using CNN-derived segmentation masks, a Detectron2 model was trained on the official CTC training data and subsequently used to infer segmentation masks for all frames, which were then provided as inputs to the proposed Tui tracker. The tracking results for the DIC-C2DH-HeLa dataset are shown in Table 4.

**Table 4.** Tracking statistics on GT and Detectron2-inferred masks from the DIC-C2DH-HeLa dataset. Best performance values are shown in bold. ILP cost: 1, 1, 10, and 20 for GT and 1, 5, 10, and 20 for Detectron 2 data (transition, mitosis, fusion, and appearance/disappearance).

CTC metrics	GT	GT mask						Detectron2 mask					
		Basic	FWD	BWD	ILP	TS	TM	Basic	FWD	BWD	ILP	TS	TM
TRA	1	0.981	0.980	0.956	**0.983**	0.980	0.629	0.970	0.971	0.951	**0.970**	**0.970**	0.655
SEG	1	0.965	0.965	0.965	**0.965**	**0.965**	0.585	0.888	0.888	0.888	0.872	**0.888**	0.441
DET	1	0.986	0.986	0.986	**0.986**	0.980	0.680	0.972	0.972	0.972	**0.972**	0.971	0.698
IDF1	1	0.762	0.781	0.532	0.865	**0.964**	0.276	0.821	0.916	0.666	0.878	**0.955**	0.346
MOTA	1	0.963	0.954	0.787	0.969	**0.976**	0.299	0.931	0.930	0.801	0.927	**0.944**	0.300
HOTA	1	0.807	0.824	0.644	0.899	**0.968**	0.372	0.840	0.920	0.737	0.891	**0.948**	0.378
CHOTA	1	0.811	0.816	0.681	0.871	**0.969**	0.313	0.822	0.917	0.771	0.896	**0.952**	0.356
LNK	1	0.951	0.944	0.751	0.966	**0.978**	0.280	0.951	0.959	0.808	0.956	**0.963**	0.360
CT	1	0.270	0.272	0.075	0.494	**0.722**	0.013	0.207	0.354	0.083	0.327	**0.487**	0
TF	1	0.801	0.826	0.602	0.881	**0.950**	0.239	0.881	0.886	0.705	0.849	**0.904**	0.241
MT	1	0.605	0.500	0.290	0.632	**0.790**	0.194	0.711	0.711	0.395	0.632	**0.763**	0.306
ML	0	0	0	0.026	0	0	0.111	0	0	0.026	0	0	0.056
IDSW	0	25	36	223	19	**5**	244	14	16	160	19	**8**	183
TP	1,120	1,104	1,104	1,104	**1,104**	1,098	943	1,093	1,093	1,093	**1,093**	1,090	859
FN	0	16	16	16	**16**	22	177	27	27	27	**27**	30	233
FP	0	0	0	0	0	0	2	35	35	35	35	**24**	233
Precision	1	1.000	1.000	1.000	1.000	**1.000**	0.998	0.969	0.969	0.969	0.969	**0.979**	0.787
Recall	1	0.986	0.988	0.988	**0.986**	0.980	0.842	0.976	0.976	0.976	**0.976**	0.973	0.767
BIO	1	0.357	0.483	0.266	0.744	**0.869**	0.084	0.363	0.649	0.314	0.627	**0.738**	0.080
OP_CLB_	1	0.654	0.714	0.508	0.855	**0.923**	0.182	0.657	0.804	0.561	0.792	**0.851**	0.220

BWD, backward; CCA, cell cycle accuracy; CT, complete track reconstruction; CTC, Cell Tracking Challenge; FN, false negative; FP, false positive; FWD, forward; GT, ground truth; IDSW, ID switching; ILP, this work; MT, mostly tracker; ML, mostly loss; FWD, forward; BWD, backward; ILP, this work; TF, track fraction; TM, TrackMate; TS; TS, TRACKASTRA; TP, true positive.

The DIC-C2DH-HeLa dataset was selected because it exhibits relatively high cell confluency (from 39.6% in the first frame to 62% in the last frame) and frequent mitotic events compared with other label-free datasets such as PhC-C2DH-U373. These characteristics create a challenging scenario for evaluating lineage reconstruction and division detection, which are key capabilities of the proposed Tui tracker. Label-free imaging modalities such as the DIC often produce segmentation masks with lower contrast and higher ambiguity than fluorescence microscopy, providing a realistic test of tracker robustness under imperfect segmentation conditions.

In both GT masks and Detectron2-derived masks, although our ILP-based method achieves the highest TRA (0.983 and 0.970, respectively), it falls behind in several identity-consistency metrics compared with TRACKASTRA, which is specifically trained on CTC datasets.

Although instance-aware segmentation of cells from label-free phase-contrast microimages using Detectron2 is very powerful, the inherent noise in neural networks often causes intermittent missing detections [26]. This intermittent loss of objects is evident from the decrease in the number of true-positive detections (1,120 in GT masks versus 1,093 in Detectron2 masks). Nevertheless, high-performance trackers such as ILP and TRACKASTRA remain capable of maintaining robust lineage reconstruction despite these segmentation errors, while Trackpy-based methods and TrackMate suffer marked performance degradation. In particular, TrackMate, which relies on a linear assignment formulation, exhibits the lowest scores across most metrics, with BIO = 0.08 and OP_CLB_ = 0.22, confirming its limited ability to reconstruct lineage relationships under realistic segmentation noise.

To complement this evaluation, we additionally analyzed the fluorescence-based Fluo-N2DH-SIM+ dataset from the CTC benchmark suite. Unlike the DIC dataset, fluorescence microscopy provides strong signal contrast and therefore more reliable segmentation masks. This dataset contains 65 frames with cell counts ranging from approximately 30 to 41 cells per FOV and includes 29 mitosis events across lineage trees extending up to 3 generations, making it particularly suitable for evaluating lineage reconstruction in proliferating populations.

The quantitative comparison between the proposed ILP-based tracker and TRACKASTRA is summarized in Table S5. Both methods achieve near-perfect detection and segmentation consistency (SEG = 1.0000 and DET = 1.0000), indicating that performance differences arise primarily from lineage association rather than object detection. Under the default parameter setting, the ILP-based framework correctly identifies 22 mitosis events compared with 21 detected by TRACKASTRA. Furthermore, using the built-in parameter optimization function of Tui, it is possible to identify parameter settings capable of recovering all 29 annotated mitosis events.

Taken together, the combined evaluation of the DIC-C2DH-HeLa and Fluo-N2DH-SIM+ datasets demonstrates that the proposed ILP-based framework performs robustly under both label-free imaging conditions with imperfect segmentation and fluorescence microscopy scenarios with dense mitotic activity.

### Tracking of the T98G electrotaxis dataset

Tracking moving cells in directional migration experiments remains a difficult task, and researchers often rely on manual tracking due to the challenge of maintaining correct cell identities over time. This manual effort frequently becomes a major bottleneck in modern single-cell migration studies. Here, the tracking performance of each method was evaluated using both human-annotated GT segmentation masks and Detectron2-generated masks for the T98G glioblastoma electrotaxis dataset [48]. The Detectron2 segmentation achieves a pixel-level intersection over union of approximately 0.76 and a Dice (F1-score) of approximately 0.87, indicating moderate but imperfect segmentation quality.

Biologically, this dataset represents a challenging tracking scenario due to relatively high cell density and directional migration induced by the electric field. Approximately 70 to 72 cells are present per FOV across 37 frames, with 8 mitosis events occurring across lineages extending up to 2 generations. Directional migration increases spatial proximity and neighbor exchange, which complicates lineage reconstruction and increases the likelihood of trajectory fragmentation.

The tracking statistics of the T98G electrotaxis dataset are summarized in Table 5. The high number of cells in the FOV substantially increases the computational cost for Trackpy-based methods, where the runtime rises from 45.22 s in basic mode to 712 s in BWD tracking mode. In contrast, the ILP framework maintains a comparable runtime while simultaneously modeling biologically meaningful events such as mitosis.

**Table 5.** Tracking statistics on GT and Detectron2-inferred masks from the T98G electrotaxis dataset. Best performance values are shown in bold.

Tracking statistics	GT	GT mask					Detectron2 mask				
		Basic	FWD	BWD	ILP	TS	Basic	FWD	BWD	ILP	TS
Analysis time (s)	2,543	**45.22**	381.06	712.04	45.21	62.8	48.24	488.63	673.81	**41.72**	56.96
Total unique tracks	101	89	125	411	93	**93**	195	287	417	204	**183**
Average track length (frames)	26.22	29.75	21.18	6.44	**28.47**	**28.47**	13.28	9.02	6.21	12.69	**13.96**
Mitosis events (2 daughters)	8	0	21	164	**8**	9	0	28	121	**8**	26
Cell count first frame	71	71	71	71	71	71	67	67	67	67	66
Cell count last frame	73	73	73	73	73	73	70	70	70	70	69
Life cycle time average (frames)	26.27	29.75	21.18	6.44	**28.47**	**28.47**	13.28	9.02	6.21	12.69	**13.96**
Life cycle time median (frames)	37	37	22	1	37	37	6	4	1	6	**9**

GT, ground truth; FWD, forward; BWD, backward; ILP, this work; TS, TRACKASTRA.

Using GT masks, both the ILP-based method and TRACKASTRA achieve similar performance and reconstruct nearly correct numbers of unique tracks (101 in GT compared with 93 in ILP and 93 in TRACKASTRA). Notably, the ILP framework correctly identifies all 8 mitosis events, matching the GT annotation, while maintaining a low number of IDSW, with only 5 switches.

Among the biologically motivated consistency metrics, the ILP-based method achieves the highest BIO (0.912) and OP_CLB_ (0.954) scores under GT masks. These results demonstrate that the proposed framework effectively preserves biologically meaningful lineage structures and accurately reconstructs mitotic branching events even in a challenging migration dataset. Table 6 summarizes the results of the CTC evaluation metrics.

**Table 6.** CTC metrics on GT and Detectron2-inferred masks from the T98G electrotaxis dataset. Best performance values are shown in bold. ILP cost: 1, 7.5, 20, and 20 for GT and 1, 18.5, 20, and 20 for Detectron2 data (transition, mitosis, fusion, and appearance/disappearance).

CTC metrics	GT	GT mask						Detectron2 mask					
		Basic	FWD	BWD	ILP	TS	TM	Basic	FWD	BWD	ILP	TS	TM
TRA	1	0.9986	0.9974	0.9809	**0.9995**	0.9994	0.767	0.923	0.92	0.912	**0.923**	0.9197	0.858
SEG	1	1	1	1	1	1	0.665	0.725	0.725	0.725	**0.725**	0.721	0.674
DET	1	1	1	1	1	1	0.817	0.931	0.93	0.931	**0.931**	0.927	0.877
IDF1	1	0.946	0.903	0.768	**0.963**	0.962	0.528	**0.808**	0.71	0.705	0.804	0.802	0.726
MOTA	1	0.997	0.985	0.8773	**0.9981**	**0.9981**	0.509	0.847	0.818	0.765	0.846	**0.850**	0.784
HOTA	1	0.964	0.929	0.829	**0.975**	0.974	0.604	0.823	0.757	0.746	0.82	**0.822**	0.753
CHOTA	1	**0.965**	0.928	0.835	0.9533	0.9539	0.603	0.822	0.76	0.767	0.8111	**0.819**	0.753
LNK	1	0.989	0.979	0.849	**0.9957**	0.9956	0.424	0.87	0.849	0.783	0.8715	**0.8721**	0.726
CT	1	0.684	0.407	0.137	0.753	**0.763**	0.066	0.169	0.098	0.07	0.164	**0.197**	0.092
TF	1	0.981	0.930	0.812	0.982	**0.985**	0.439	0.787	0.728	0.722	0.785	**0.789**	0.619
MT	1	0.97	0.881	0.713	0.96	**0.97**	0.41	0.04	0.04	0.04	0.04	0.04	0.61
ML	0	0	0	0.02	0	0	0.09	0	0	0	0	0	0
IDSW	0	3	39	325	**5**	**5**	439	79	157	302	83	**81**	147
TP	2,648	2,648	2,648	2,648	**2,648**	**2,648**	2,528	2,500	2,499	**2,500**	**2,500**	2,487	2,409
FN	0	0	0	0	0	0	120	148	149	148	**148**	161	239
FP	0	0	0	0	0	0	10	133	133	133	133	112	**18**
Precision	1	1	1	1	1	1	0.996	0.95	0.95	0.95	0.950	0.957	**0.993**
Recall	1	1	1	1	1	1	0.955	0.944	0.944	0.944	**0.944**	0.939	0.901
BIO	1	0.555	0.584	0.34	**0.912**	0.896	0.168	0.319	0.331	0.279	0.400	**0.407**	0.237
OP_CLB_	1	0.772	0.781	0.594	**0.954**	0.946	0.296	0.595	0.59	0.531	0.636	**0.640**	0.482

BWD, backward; CCA, cell cycle accuracy; CT, complete track reconstruction; CTC, Cell Tracking Challenge; FN, false negative; FP, false positive; FWD, forward; GT, ground truth; IDSW, ID switching; ILP, this work; MT, mostly tracker; ML, mostly loss; FWD, forward; BWD, backward; ILP, this work; TF, track fraction; TM, TrackMate; TS; TS, TRACKASTRA; TP, true positive.

When tracking is performed using Detectron2-generated masks, the number of reconstructed tracks increases substantially (204 and 183 tracks for ILP and TRACKASTRA, respectively), indicating trajectory fragmentation caused by imperfect segmentation. This comparison between GT and learned segmentation provides a direct evaluation of tracking robustness under realistic segmentation degradation conditions. Such fragmentation interferes with automated mitosis detection, as segmentation artifacts or cell overlaps may be misinterpreted as division events. For example, in TRACKASTRA, the number of detected mitosis events increases from 9 under GT masks to 26 under Detectron2 segmentation, highlighting the sensitivity of mitosis detection to segmentation-induced artifacts.

Importantly, the impact of segmentation degradation can be partially mitigated through the optimization of the ILP cost weights. As further demonstrated in the sensitivity analysis (Table S4, described in the following section), appropriate tuning of mitosis and fusion penalties ($w_{m}$ and $w_{f}$) substantially reduces spurious event detection and improves biologically relevant metrics such as BIO and OP_CLB_, even under imperfect segmentation. This demonstrates that segmentation-induced errors are not only observable but can also be corrected within the proposed optimization framework, highlighting both robustness to segmentation noise and adaptability through parameter calibration to recover meaningful lineage structures.

### Hyperparameter-sensitivity analysis

**Fig. 3.**
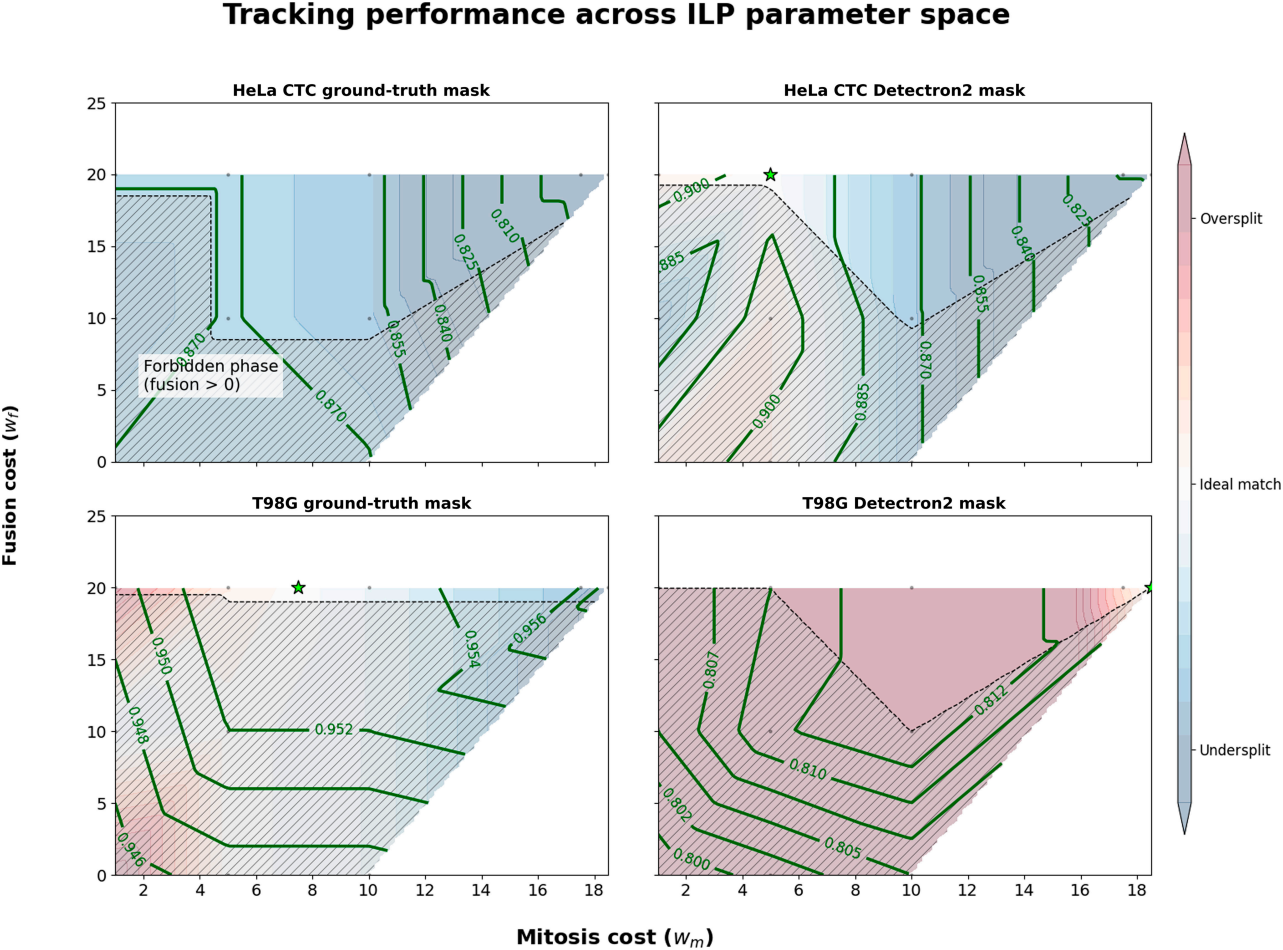
Topological phase diagrams mapping tracking accuracy (TRA) across the integer linear programming (ILP) cost parameter space. Phase diagrams show the TRA performance as a function of mitosis cost ($w_{m}$) and fusion cost ($w_{f}$) for HeLa (top row) and T98G (bottom row) datasets using ground truth segmentation masks (left column) and Detectron2-generated masks (right column). Contour lines denote cell-specific higher-order TRA values; star markers indicate optimal parameter combinations for the dataset. CTC, Cell Tracking Challenge.

To evaluate the contribution of the ILP cost weights to tracking performance and assess the robustness of our default parameter configuration, we conducted a systematic sensitivity analysis varying the mitosis cost weight ($w_{m}\in \left[1,20\right]$) and fusion cost weight ($w_{f}\in \left[0,25\right]$) while keeping other parameters fixed at their default values (Table S4). We constructed topological phase diagrams mapping CHOTA TRA across this parameter space for 4 experimental conditions: HeLa and T98G datasets with either GT or Detectron2-generated segmentation masks (Fig. 3).

The phase diagrams reveal 3 distinct regimes governing tracking behavior. An oversplit phase (red regions) emerges when mitosis costs are insufficiently penalized, causing the optimizer to favor spurious division events that fragment continuous tracks. Conversely, an undersplit phase occurs when mitosis costs are excessively high, leading to missed mitosis events and artificially merged trajectories, which may be incorrectly interpreted as fusion events. Between these extremes lies an optimal matching region (white), where tracking performance is maximized. The diagonal hatched region indicates a forbidden phase where fusion becomes favorable relative to mitosis ($w_{f}<w_{m}$), resulting in biologically implausible solutions for proliferating cell populations.

With GT segmentation masks, both datasets exhibit broad plateaus of high accuracy that are robust to parameter variation, with T98G achieving CHOTA > 0.95 and HeLa reaching CHOTA > 0.87 across wide parameter ranges (Table S4). This stability indicates that the ILP framework is inherently robust under high-quality segmentation conditions and does not rely on narrow parameter tuning.

In contrast, when using Detectron2-generated masks, the optimal parameter region shifts and becomes more constrained. This effect is particularly pronounced in the T98G dataset, where segmentation errors induce a transition toward the undersplit regime, characterized by missed mitosis events and increased false fusion interpretations. This contraction of the optimal parameter region highlights the impact of segmentation quality on tracking performance. It also suggests that weakly supervised parameter calibration, guided by simple biological priors (e.g., approximate cell count or number of mitosis events), can help recover optimal performance under imperfect segmentation.

Biologically motivated metrics (BIO and OP_CLB_) show greater sensitivity to cost weights than TRA (Table S4), reflecting their dependence on accurate event detection. Across all conditions, the ILP-based optimization consistently outperforms the Trackpy baseline without global optimization (e.g., BIO improves from 0.357 to 0.744 for HeLa GT masks). The default parameter configuration ($w_{m}=5$ and $w_{f}=20$; indicated by star markers in Fig. 3) represents a robust compromise, achieving near-optimal performance across datasets without condition-specific tuning.

The relative stability of TRA compared with BIO and OP_CLB_ suggests that cost weights primarily influence biological event detection rather than basic trajectory linking. This observation supports the biological interpretability of the ILP framework: Mitosis and fusion costs regulate competing lineage hypotheses, where higher penalties suppress spurious events, while moderate values allow genuine biological events to be correctly captured.

## Discussion

Single-cell tracking has been approached using diverse methodological paradigms [51]. In contrast to Usiigaci [26], which relies on Trackpy for one-to-one linking and scales as $O\left(N\cdot C\right)$ by considering only simple transitions, our ILP framework explicitly incorporates mitosis and fusion through event-specific hypotheses. While this additional modeling increases computational complexity, it enables the reconstruction of lineage relationships that heuristic linkers cannot capture (see the Supplementary Materials for a detailed complexity analysis).

From a complexity perspective, our ILP method scales as $O\left(N\cdot C^{2}\right)$, which is heavier than assignment-based strategies such as the DeepCell family [33], which use the Hungarian algorithm [$O\left(C^{3}\right)$ per frame] but do not explicitly model lineage events. However, it remains asymptotically lighter than transformer-based trackers [17,36], which scale as $O\left(N^{2}\right)$ due to self-attention and require substantial graphics processing unit memory for long sequences.

In practice, asymptotic complexity does not directly predict wall-clock runtime. We observed that runtime depends strongly on dataset characteristics, such as sequence length and cell density, as well as implementation and hardware factors. For example, on the DIC-C2DH-HeLa dataset (84 frames and ∼12 cells in the first frame), the ILP framework achieved a runtime of 5.37 s compared to 13.03 s for TRACKASTRA. On the Fluo-N2DH-SIM+ dataset (64 frames and ∼30 cells), runtimes were 20.67 and 28.62 s, respectively. In contrast, on long synthetic sequences with higher combinatorial complexity, the transformer-based implementation processed the dataset faster due to graphics processing unit parallelization. These results indicate that practical efficiency depends not only on theoretical scaling but also on dataset size, solver behavior, and hardware acceleration. Therefore, general claims about computational efficiency should be interpreted with caution.

Prior global lineage-tracking frameworks, including the chain-graph model [52], graphical models for dividing cells [21], and moral lineage tracing formulations [53], as well as more recent ILP-based trackers such as Ultrack [28], formulate tracking as a global optimization problem under biologically valid lineage constraints. However, these formulations typically model 1-to-1 transitions and 1-to-2 mitotic divisions, while multiparent relationships are not explicitly represented, and apparent merges are often treated as segmentation artifacts. In contrast, the Tui framework introduces 2-to-1 fusion hypotheses as explicit events within the optimization. By assigning dedicated variables and costs to fusion events, the solver jointly evaluates merging alongside transition, appearance/disappearance, and mitosis events, enabling the reconstruction of lineage structures that include both splitting and merging dynamics.

Our experimental evaluation shows that the ILP-based framework achieves competitive performance while offering advantages in biological interpretability and deployment flexibility. Under GT conditions, the method attains near-perfect tracking metrics, indicating that global optimization effectively enforces temporal and lineage consistency. In more challenging scenarios involving rapid migration or irregular cell behavior, such as those in the T98G dataset, parameter calibration becomes increasingly important. Compared with learning-based approaches, which often require extensive hyperparameter tuning and annotated training data [16,36], the Tui framework enables biologically interpretable calibration using macroscopic constraints such as total cell count, number of mitosis events, or number of fusion events.

Several potential failure modes warrant consideration. While deep-learning techniques such as CNNs and transformers have provided powerful solutions for segmentation, instance segmentation of whole cells and subcellular components without staining, particularly under noisy and densely populated conditions, remains challenging [6,26]. Imperfect segmentation may lead to merged or fragmented detections, which can be incorrectly interpreted as fusion or mitosis events, resulting in lineage reconstruction errors [54]. In densely populated regions, multiple biologically plausible associations may exist, and the global optimizer may select a solution that is optimal with respect to the cost function but not necessarily biologically correct. Similarly, large cell displacements or missed detections can prevent correct edge construction, limiting the ability of the ILP to recover true trajectories. Transient cell overlap or segmentation artifacts may also be misinterpreted as fusion events, particularly in the absence of additional biological constraints. While the ILP framework demonstrates robustness to moderate segmentation noise and allows partial recovery of biologically meaningful lineage structures through cost parameter optimization, its performance remains fundamentally constrained under severe segmentation degradation. This motivates the design of the expert-correctable interface in Tui, which enables users to efficiently inspect, validate, and correct lineage structures when automated inference is uncertain.

A potential concern is the risk of dataset-specific parameter tuning. To address this, we conducted a sensitivity analysis by systematically varying the mitosis ($w_{m}$) and fusion ($w_{f}$) costs across a wide range (Table S4). The results show a broad plateau of stable performance across parameter configurations, with minimal variation in both TRA and biological consistency metrics (BIO and OPCLB). This suggests that the ILP formulation is not highly sensitive to precise parameter values and does not rely on narrow dataset-specific tuning.

Another limitation is computational cost. The global optimization strategy introduces additional overhead compared with greedy or frame-by-frame methods, particularly for large-scale datasets with high cell density or long temporal sequences. While this cost is manageable for typical biological experiments, further optimization or parallelization may be required for large-scale or real-time applications.

Finally, the ability to model cell fusion represents a unique feature of the proposed framework. Although fusion events are relatively rare [55], they play critical roles in processes such as myogenesis, immune response, and tissue development [6,23]. While such events were not present in the real datasets analyzed here, incorporating fusion into the lineage model extends the applicability of the framework to biological systems where merging dynamics are essential.

Future work will focus on validating this capability using experimentally annotated fusion datasets and extending the framework to more complex imaging modalities.

## Conclusion

This work presents the Tui tracker, a comprehensive cell-tracking framework that addresses fundamental limitations in current methodologies through algorithmic innovations and user-oriented considerations. Our ILP algorithm enables global optimization across temporal sequences while maintaining computational efficiency. Our lineage-aware, expert-correctable mechanisms provide enhanced robustness in challenging scenarios. Most notably, our framework uniquely supports cell fusion event detection alongside accurate division timing identification, capabilities that remain largely absent in other SOTA tracking systems.

The comprehensive experimental validations of the synthetic migration dataset, the T98G brain glioblastoma cell line electrotaxis dataset, and publicly available CTC datasets confirm the practical effectiveness of our approach. Under ideal segmentation conditions, the proposed method achieves near-perfect TRA (0.98 in the DIC-C2DH-HeLa dataset, 0.99 in the Fluo-N2DH-SIM+ dataset, and 0.99 in the T98G dataset) while maintaining competitive runtime performance compared with existing SOTA tracking approaches.

Under realistic conditions with intermittent segmentation errors, the Tui tracker still shows strong robustness and achieves higher scores on biologically meaningful lineage metrics such as BIO and OPCLB. These results demonstrate that global optimization with explicit modeling of biological events can improve lineage reconstruction while maintaining practical usability for experimental datasets. The consistent performance in biologically relevant metrics, combined with the interpretable optimization formulation and the absence of training data requirements, highlights the value of the Tui tracker for time-lapse quantitative biology studies where lineage reconstruction and automated cellular dynamics analysis are required.

The user-friendly GUI and expert-correction interface further enable experimental users to validate and refine lineage graphs efficiently, reducing the manual effort typically associated with long time-lapse tracking experiments.

Currently, the Tui tracker focuses on 2-dimensional time-lapse microscopy datasets, which represent the most common format in many live-cell imaging studies. Support for multichannel imaging and 3-dimensional object tracking will be incorporated in the future to enable lineage reconstruction in volumetric datasets and whole-organism imaging applications, further extending its utility in developmental biology and tissue-level studies.

Furthermore, deep-learning-based feature extraction methods have been promising for multimodal computational imaging, subcellular segmentation, and phenotype prediction [56–58]. Integrating such feature representations with the lineage-aware tracking capabilities of Tui may enable the automated analysis of subcellular dynamics, population behavior, and cellular phenotypes across time-lapse experiments. These developments may further advance quantitative cell biology, cancer research, and high-throughput screening applications where understanding lineage relationships and dynamic cellular behavior is essential.

## Data Availability

All the example datasets used in this study, including synthetic datasets, segmentation masks, curated lineage annotations, and example tracking results, are publicly available on Zenodo at https://zenodo.org/records/19026908.

## Supplementary Materials

Supplementary 1 Figs. S1 and S2 Sections S1 to S3 Tables S1 to S5 Reference [59]
