# Supplementary material for: Tui: A Multigenerational and Expert-Correctable Tracker for Cellular Dynamics
Source: Comput Struct Biotechnol J. 2026 Apr 24;35(1):0054. doi: 10.34133/csbj.0054 (PMC13106940; doi:10.34133/csbj.0054)
Supplement: Supplementary 1 — Figs. S1 and S2 Sections S1 to S3 Tables S1 to S5 Reference [59] [file csbj.0054.f1.pdf]

Tui: A Multi-generational and Expert-correctable Tracker for Cellular Dynamics

I-Ming Chang and Hsieh-Fu Tsai

1 Detailed Computational Complexity Analysis of trackpy-based Algorithm

1.1 Complete Algorithm Specifications

The forward and backward tracking algorithm consists of four main components implemented as detailed algorithmic procedures:

Algorithm 1: Cell Tracking - Part 1: Feature Extraction

Input: 3D mask array M, tracking mode
Output: Features dataframe F

Initialize parameters: $n_{features} = 8$, weights W, global ID counter;
if *mode == "backward"* **then**
  Process frames $T_i \to T_0$ (reverse);
else
  Process frames $T_0 \to T_i$ (forward);
end
for *each frame t* **do**
  Extract regions using `regionprops`($M[:, :, t]$);
  for *each region r* **do**
    Compute features: centroid, area, diameter, etc.;
    Calculate weighted features: $f_{\text{weighted}} = W \cdot f_{\text{original}}$;
  end
end
Sort features by frame and **return** F;

Algorithm 2: Cell Tracking - Part 2: Trajectory Linking

Input: Features F, parameters (sr, mem)
Output: Initial trajectories $\mathcal{T}$

Prepare position columns for linking;
Validate input data (check for missing values, empty frames);
$\mathcal{T} \leftarrow$ `tp.link`(features, search_range=sr, memory=mem);
Initialize daughter ID counter: $next\_id \leftarrow \max(\mathcal{T}.\text{particle}) + 1$;
return $\mathcal{T}$ *with particle IDs*;

Algorithm 3: Cell Tracking - Part 3: Division/Fusion Detection

```
Input: Trajectory T, detection parameters
Output: Updated T with parent-child relationships
if mode == "forward" then
    // Forward: Detect mitosis events
    for each frame transition t_i → t_{i+1} do
        for each parent P in frame t_i do
            Find best daughter pair (D_1, D_2) in frame t_{i+1};
            if mitosis criteria satisfied then
                Create new daughter IDs and update relationships;
                Split tracks at mitosis point;
            end
        end
    end
else
    // Backward: Detect fusion events
    for each frame transition t_{i+1} → t_i (reverse) do
        for each parent P in frame t_i do
            Find best daughter pair (D_1, D_2) in frame t_{i+1};
            if fusion criteria satisfied then
                Create new daughter IDs and update relationships;
            end
        end
    end
end
return T with division/fusion events;
```

Algorithm 4: Cell Tracking - Part 4: Validation and Finalization

```
Input: Updated trajectory T
Output: Final trajectory with lineage map
// Validation
Check for self-parenting, orphaned children, temporal consistency;
// Re-indexing
Sort particles by (frame, y, x, particle);
Create sequential ID mapping: old_id → new_id;
Apply mapping to particle and parent_particle columns;
// Build Lineage
lineage_map ← group children by parent ID;
Sort final trajectory and reset index;
return T_final, lineage_map;
```

Algorithm 5: Division/Fusion Criteria Validation

Input: Parent P, daughters (D_1, D_2), thresholds
Output: Boolean: valid event

`// Distance Check`
if $||P - D_i||_2 > P.diameter \times factor_{dist}$ *for any* D_i **then**
  return *false*;
end
`// Area Check`
$ratio = \frac{D_1.\text{area}+D_2.\text{area}}{P.\text{area}}$;
if $ratio \notin [factor_{min}, factor_{max}]$ **then**
  return *false*;
end
`// Similarity Check`
$sim = \frac{\min(D_1.\text{area}, D_2.\text{area})}{\max(D_1.\text{area}, D_2.\text{area})}$;
if $sim \leq threshold_{sim}$ **then**
  return *false*;
end
return *true*;

To provide comprehensive evaluation benchmarks and accommodate diverse experimental requirements, the framework additionally implements three heuristic tracking methodologies: Basic, Forward, and Backward modes (Fig. S1). These approaches utilize rule-based trajectory linking algorithms coupled with pattern-based mitosis detection mechanisms, operating without the computational overhead of the complete ILP optimization procedure. While these heuristic modes serve as important baseline comparisons and may prove suitable for specific experimental contexts with relaxed accuracy requirements, the ILP-based optimization module constitutes the principal methodological contribution of this work. Empirical analysis demonstrates that despite the global optimization complexity inherent in the ILP formulation, the proposed method achieves computational efficiency comparable to or superior to the heuristic alternatives while consistently delivering substantially improved tracking accuracy and lineage reconstruction fidelity.

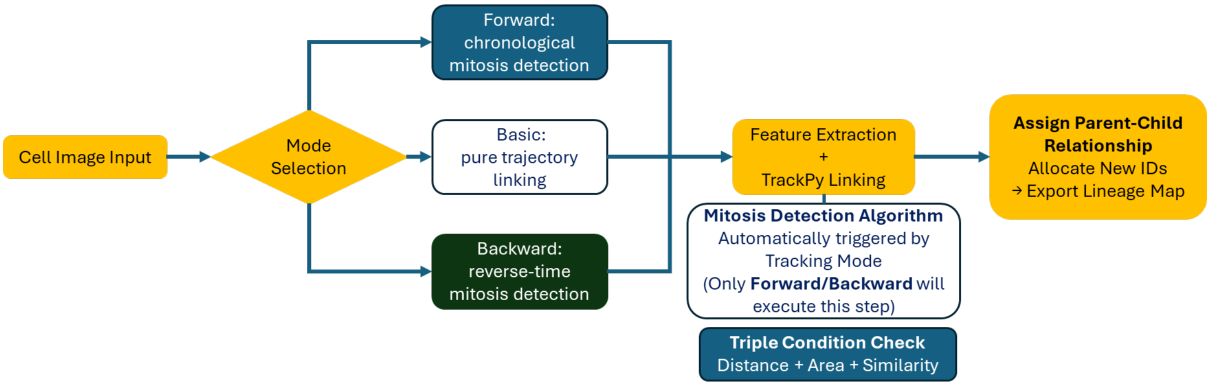

Fig. S1: Three different tracking modes. Temporal linking strategies are shown: the Basic mode performs straightforward frame-to-frame association, the Forward mode incorporates predictive trajectory estimation, and the Backward mode leverages retrospective information to refine associations.

1.2 Computational Complexity Analysis

Let F be the number of frames, C the average number of cells per frame, and $N = F \cdot C$ the total number of detected cell instances. The detailed complexity analysis for each component is as follows:

- **Feature Extraction (Algorithm 1):** $O(N)$ time and space complexity. Each frame is processed independently using `regionprops`, with linear scaling in the number of detected regions. Feature computation and weighting operations are performed in constant time per cell.
- **Trajectory Linking (Algorithm 2):** $O(N \log N)$ time complexity, as *Trackpy* employs spatial indexing structures (e.g., KDTree) for efficient nearest neighbor search during linking. Space complexity remains $O(N)$ for storing trajectory information.
- **Mitosis/Fusion Detection (Algorithm 3):** This component dominates the overall runtime with $O(F \cdot C^3) = O(N \cdot C^2)$ time complexity in the worst case. For each frame transition, all possible combinations of parent and daughter cell pairs must be evaluated, leading to cubic scaling with the number of cells per frame.
- **Validation and Finalization (Algorithm 4):** Sorting and mapping operations contribute $O(N \log N)$ time complexity. Lineage construction requires grouping operations that scale linearly with the number of parent-child relationships.

$$\text{Overall Time Complexity} = O(N \cdot C^2) \tag{1}$$

$$\text{Overall Space Complexity} = O(N) \tag{2}$$

The feature extraction and linking components contribute $O(N \log N)$ time complexity but are asymptotically dominated by the event detection step when the average number of cells per frame (C) becomes large, which is typical in dense cell populations.

TABLE S1: Time and Space Complexity of Algorithm Components

**Component**	**Algorithm**	**Time Complexity**	**Space Complexity**
Feature Extraction	Algorithm 1	$O(N)$	$O(N)$
Trajectory Linking	Algorithm 2	$O(N \log N)$	$O(N)$
Event Detection	Algorithm 3	$O(N \cdot C^2)$	$O(N)$
Validation & Finalization	Algorithm 4	$O(N \log N)$	$O(N)$
**Overall System**	**Combined**	$O(N \cdot C^2)$	$O(N)$

1.3 Implementation Details and Parameter Configuration

Feature Weighting Strategy: The weighting scheme emphasizes spatial coordinates and morphological features differently based on their reliability and biological significance. Centroid positions receive the highest weights as they provide the most reliable tracking signals, while morphological features are weighted based on their stability across frames.

Validation Procedures: The algorithm incorporates multiple validation steps to ensure biological plausibility:

- **Self-parenting check:** Prevents cells from being assigned as their own parents
- **Orphaned children detection:** Identifies daughter cells without valid parent assignments
- **Temporal consistency validation:** Ensures parent-child relationships respect temporal ordering
- **Duplicate relationship elimination:** Removes redundant parent-child assignments

Parameter Sensitivity: The threshold parameters α_{dist}, β_{min}, β_{max}, and γ_{sim} require careful tuning based on experimental conditions. Typical ranges are: $\alpha_{\text{dist}} \in [1.5, 3.0]$, $\beta_{\text{min}} \in [0.7, 1.0]$, $\beta_{\text{max}} \in [1.3, 2.0]$, and $\gamma_{\text{sim}} \in [0.3, 0.7]$, with specific values dependent on cell type and imaging conditions.

Memory and Search Range Configuration: The *trackpy* parameters require adjustment based on cell motility and imaging frequency. Search range should accommodate maximum expected displacement between frames, while the memory parameter should account for temporary occlusions or segmentation failures that may cause cells to disappear for short periods.

2 Detailed Computational Complexity Analysis of ILP-based Algorithm

We analyze the time and space complexity of our ILP-based tracking pipeline. Let N denote the total number of detected cell instances across all frames, F the number of frames, and C the average number of cells per frame (i.e., $N \approx F \cdot C$).

- **Node Extraction**: This step iterates over each frame and each label within it to extract region properties. Using `skimage.regionprops`, it has a time complexity of $O(N)$ and space complexity of $O(N)$ to store node features.
- **Edge Construction**:
  - *Transition Edges:* For each pair of adjacent frames, all pairs of cells are compared using Euclidean distance, resulting in $O(C^2)$ per frame or $O(F \cdot C^2) = O(N \cdot C)$ total time.
  - *Mitosis Edges:* For each parent in frame t, all close daughter pairs in frame $t+1$ are evaluated. This yields $O(C^3)$ per frame or $O(F \cdot C^3) = O(N \cdot C^2)$ time complexity.
  - *Fusion Edges:* Similar to mitosis, all close parent pairs to each candidate in frame $t+1$ are evaluated. This also leads to $O(N \cdot C^2)$ time.

  The space complexity for edge construction is $O(E)$, where E is the number of valid edges ($E \ll N^2$ in practice due to distance filtering).
- **ILP Formulation and Solving**:
  - The number of binary variables is $O(E + 2N)$ (edges + appearance + disappearance).

- The number of constraints is $O(N)$ due to flow conservation.
- Solving MILP is NP-hard in theory, but commercial solvers (e.g., Gurobi) or SciPy's *milp()* handle moderate problem sizes efficiently.
- Worst-case time complexity is exponential in $E + N$, but average-case performance is acceptable up to thousands of nodes.

- **Trajectory Reconstruction**: Building lineage and assigning track IDs involves single-pass traversal of the graph and sorting. The time complexity is $O(N + E)$, and space is $O(N)$.

Overall, the runtime is dominated by the edge construction phase — specifically mitosis and fusion handling — resulting in a worst-case time complexity of $O(N \cdot C^2)$ and space complexity of $O(N + E)$.

TABLE S2: Time and Space Complexity of Key Components

Component	Time Complexity	Space Complexity
A	$O(N)$	$O(N)$
B	$O(N \cdot C)$	$O(E)$
C	$O(N \cdot C^2)$	$O(E)$
D	$O(N \cdot C^2)$	$O(E)$
E	$O(N + E)$	$O(N + E)$
F	Exponential in $N + E$ (worst case)	$O(N + E)$
G	$O(N + E)$	$O(N)$

Component Descriptions:

A. Extract nodes (cell detections) from all frames.

B. Build 1-to-1 transition hypotheses between frames.

C. Generate all valid 1-to-2 mitosis hypotheses.

D. Generate all valid 2-to-1 fusion hypotheses.

E. Set up MILP objective and constraints.

F. Solve MILP using Gurobi or SciPy solver.

G. Parse selected edges to reconstruct trajectories and lineage.

2.1 Event Cost Functions

To integrate biological plausibility into the ILP, we explicitly defined cost functions for transitions, mitosis, and fusion. Each function combines geometric displacement with approximate size conservation, reflecting the assumption that cell motion is smooth and that mitotic and fusion events preserve area at the population level.

$$f_{\text{trans}}(v_i^t, v_j^{t+1}) = \alpha \cdot \|c_i^t - c_j^{t+1}\|_2 + \beta \cdot \frac{|A_i^t - A_j^{t+1}|}{A_i^t},$$

where c denotes the centroid and A the cell area. This cost penalizes large centroid displacements and relative size changes, favoring smooth transitions between consecutive frames.

$$f_{\text{mit}}(v_i^t, v_j^{t+1}, v_k^{t+1}) = \left\| c_i^t - \frac{c_j^{t+1}+c_k^{t+1}}{2} \right\|_2 + \gamma \cdot \left| \frac{A_i^t}{A_j^{t+1} + A_k^{t+1}} - 1 \right|,$$

which favors mitosis when the parent centroid aligns with the midpoint of two daughters and the combined area of the daughters matches that of the parent.

$$f_{\text{fus}}(v_i^t, v_j^t, v_k^{t+1}) = \left\| c_k^{t+1} - \frac{c_i^t+c_j^t}{2} \right\|_2 + \eta \cdot \left| \frac{A_i^t + A_j^t}{A_k^{t+1}} - 1 \right|,$$

which encourages fusion when the child centroid lies between two candidate parents and its area is consistent with their combined size.

In all three cases, the local coefficients $\alpha, \beta, \gamma, \eta$ control the relative contributions of displacement versus size conservation, while the global weights w_t, w_m, w_f govern the frequency with which each event type is selected in the overall optimization (TABLE S3). In this way, mitosis and fusion explicitly enter the ILP formulation through both their dedicated cost functions and their global weights, rather than being treated as heuristic post-processing.

TABLE S3: The ILP parameter search space

**Parameter**	**Symbol**	**Range**
Transition cost weight	w_t	[0.1, 15.0]
Mitosis cost	w_m	[5.0, 250.0]
Fusion cost	w_f	[5.0, 250.0]
Appearance cost	w_a	[10.0, 500.0]
Disappearance cost	w_d	[10.0, 500.0]
Maximum link distance	$d_{\max}$	[20, 150]

3 Detailed Evaluation Metrics Specifications

True Positives (TP), False Positives (FP), False Negatives (FN): Following the Cell Tracking Challenge (CTC) evaluation protocol[1, 2], the matching between predicted and ground truth cell instances is defined based on pixel-wise overlap. Each predicted detection is represented as a pixel set S_c, and each ground truth cell instance as a pixel set R_c, where the subscript c indexes a candidate match. A match is considered a true positive (TP) if the intersection of these two sets exceeds 50% of the ground truth region size:

$$\text{TP} = \{c \mid |S_c \cap R_c| > 0.5 \cdot |R_c|\} \tag{3}$$

This criterion ensures that each ground truth cell R_c is matched to at most one predicted cell S_c, though a single prediction may potentially be matched to multiple ground truth instances (e.g., in cases of delayed mitosis detection). False positives (FP)

are unmatched predicted cells, while false negatives (FN) are ground truth cells that have no matched prediction under the 50% overlap rule[2].

Precision and Recall:

$$\text{Precision} = \frac{\text{TP}}{\text{TP} + \text{FP}}, \quad \text{Recall} = \frac{\text{TP}}{\text{TP} + \text{FN}}$$

Segmentation Accuracy (SEG): The average Jaccard index between predicted and ground truth masks:

$$\text{SEG} = \frac{1}{|\text{TP}| + |\text{FN}|} \sum_{c \in \text{TP}} \frac{|R_c \cap S_c|}{|R_c \cup S_c|} \tag{4}$$

Tracking Accuracy (TRA): Measures the similarity between the predicted and ground truth tracking graph using the Acyclic Oriented Graph Measure (AOGM):

Acyclic Oriented Graph Measure (AOGM): To quantify tracking performance from a graph-theoretic perspective, the AOGM metric calculates the minimal cost of transforming a predicted tracking graph into the ground truth graph. Each tracking result is modeled as a directed acyclic graph where vertices represent individual cell instances and edges represent temporal associations or parent-child (mitotic) relationships. The total transformation cost is a weighted sum of six basic operations:

$$\text{AOGM} = w_{\text{NS}} \cdot \text{NS} + w_{\text{FN}} \cdot \text{FN} + w_{\text{FP}} \cdot \text{FP} + w_{\text{ED}} \cdot \text{ED} + w_{\text{EA}} \cdot \text{EA} + w_{\text{EC}} \cdot \text{EC} \tag{5}$$

where:

- NS: number of split operations (one detection matched to multiple annotations),
- FN / FP: false negatives / false positives (missed or extra detections),
- ED / EA: deletion or addition of edges (temporal links),
- EC: edge type correction (e.g., incorrect mitosis labeling).

Each error type is assigned a manually defined weight w reflecting the editing effort to correct it. A lower AOGM value indicates better tracking accuracy.

$$\text{TRA} = 1 - \frac{\min(\text{AOGM}, \text{AOGM}_0)}{\text{AOGM}_0} \tag{6}$$

where AOGM is the cost of transforming the predicted graph to ground truth, and AOGM_0 is the cost of constructing the ground truth graph from scratch.

Detection Accuracy (DET): The DET metric quantifies detection performance independently of tracking accuracy by isolating node-level operations from the full AOGM-based TRA score. Specifically, DET evaluates the cost of transforming the set of predicted detections into the set of ground truth detections, without considering linking or parent-child relationships. To achieve this, DET explicitly excludes all edge-related penalties in the AOGM formulation, i.e., $w_{\text{ED}} = w_{\text{EA}} = w_{\text{EC}} = 0$, where ED, EA, and EC represent edge deletion, addition, and correction errors, respectively. It is computed as:

$$\text{DET} = 1 - \frac{\min(\text{AOGM-D}, \text{AOGM-D}_0)}{\text{AOGM-D}_0} \tag{7}$$

where AOGM-D represents the cost of transforming the set of predicted nodes (cells) into the set of ground truth nodes, and AOGM-D_0 is the cost of generating the ground truth graph from scratch (i.e., assuming an empty prediction). The min operation in the numerator ensures the DET score remains non-negative, even when the transformation cost exceeds the baseline construction cost. This metric is conceptually similar to an F3-score, which emphasizes recall over precision—an important property in time-lapse imaging where missing cells is more detrimental than over-segmentation[3].

Linking Accuracy (LNK): The **linking accuracy** (LNK) quantifies how precisely each tracked object is temporally associated across consecutive frames. It evaluates the similarity between the acyclic oriented graph (AOG) generated by the algorithm and the reference graph derived from the ground truth annotations.

To compute LNK, two intermediate acyclic oriented graphs are constructed using synchronized vertex sets. These graphs are compared using a cost-based transformation metric with zero penalty on vertex assignment. **Similar to how DET excludes linking errors, LNK focuses solely on association quality by ignoring vertex-related errors (i.e.,** $w_{\text{NS}} = w_{\text{FN}} = w_{\text{FP}} = 0$)[2].

Formally, LNK is computed as a normalized AOGM-A (Acyclic Oriented Graph Matching for Association) score:

$$\text{LNK} = 1 - \frac{\min(\text{AOGM-A}, \text{AOGM-A}_0)}{\text{AOGM-A}_0} \tag{8}$$

where **AOGM-A** is the cost of transforming the predicted graph into the ground truth association graph, and **AOGM-A_0** represents the cost of constructing the ground truth association graph from scratch (i.e., using only vertices and no edges).

The minimum operator in the numerator ensures the LNK score remains non-negative, even when constructing the ground truth graph from scratch would require fewer operations than transforming the predicted one.

MOTA (Multiple Object Tracking Accuracy): Combines false positives, false negatives, and ID switches:

$$\text{MOTA} = 1 - \frac{\text{FN} + \text{FP} + \text{IDSW}}{\text{GT}} \tag{9}$$

IDF1 Score: Measures the ratio of correctly identified detections over the average number of ground-truth and predicted detections, emphasizing ID consistency.

ID Switches (IDSW): Number of instances where a tracked ID changes while tracking the same object.

Mostly Tracked (MT) and **Mostly Lost (ML)** are trajectory-level metrics commonly used in multi-object tracking benchmarks [4]. Each ground truth trajectory is evaluated based on the proportion of its lifespan that is successfully recovered by the tracker, regardless of ID consistency. A trajectory is considered mostly tracked (MT) if it is correctly recovered for at least 80% of its total length, and mostly lost (ML) if it is recovered for less than 20% of its lifespan. All remaining cases are categorized as partially tracked. A high MT and low ML ratio are indicative of strong tracking performance. As per standard convention, MT and ML are reported as the fraction of total ground truth trajectories falling into each category.

HOTA (Higher Order Tracking Accuracy) [5]: A unified tracking metric that balances detection and association accuracy. It is defined as:

$$\mathrm{HOTA}_\alpha = \sqrt{\frac{1}{|\mathrm{TP}| + |\mathrm{FN}| + |\mathrm{FP}|} \sum_{c \in \mathrm{TP}} A(c)} \tag{10}$$

One major advantage of HOTA is its ability to *separately quantify* detection and association quality within a unified framework. This separation allows researchers to diagnose whether performance degradation stems from poor object detection (e.g., missing or spurious segments) or from incorrect temporal linking (e.g., identity switches or fragmented tracks).

CHOTA (Cell-specific HOTA with Lineage Awareness) [2]: An extension of HOTA that incorporates mitosis-aware lineage structure. It redefines the concept of trajectory using a lineage-aware similarity function $\sigma(i, j)$:

$$\sigma(i, j) = \begin{cases} 1, & \text{if } i = j \text{ or } i \text{ is an ancestor or descendant of } j \\ 0, & \text{otherwise} \end{cases} \tag{11}$$

Both HOTA and CHOTA rely on per-object association accuracy, defined in a unified form as:

$$A^{(*)}(c) = \frac{|\mathrm{TPA}^{(*)}(c)|}{|\mathrm{TPA}^{(*)}(c)| + |\mathrm{FNA}^{(*)}(c)| + |\mathrm{FPA}^{(*)}(c)|} \tag{12}$$

where superscript $(*)$ denotes the context: for standard HOTA, we use $A^{(*)}(c) = A(c)$; for CHOTA, $A^{(*)}(c) = A_\sigma(c)$ with lineage-aware associations. Accordingly, CHOTA is defined as:

$$\mathrm{CHOTA}_\alpha = \sqrt{\frac{1}{|\mathrm{TP}| + |\mathrm{FN}| + |\mathrm{FP}|} \sum_{c \in \mathrm{TP}} A_\sigma(c)} \tag{13}$$

Complete Tracks (CT)[2]: Measures the fraction of ground truth tracks that are completely and correctly reconstructed by the prediction. It reflects how many trajectories were recovered in their entirety without ID switching or fragmentation.

Largest Track Fraction (TF)[2]: A relaxed version of CT that quantifies the largest continuously tracked portion of each ground truth trajectory, averaging over all ground truth tracks.

Branching Correctness (BC)[2]: This metric evaluates the performance of mitosis (branching event) detection by calculating the F1 score over all predicted mitosis events within a temporal tolerance of i frames. A branching event is considered correctly detected if both the predicted and ground truth tracks exhibit mitosis in approximately the same frame (within $\pm i$). The metric is computed using the following counts:

- **BTP(i)**: number of correctly matched mitosis events,
- **BFN(i)**: number of ground truth mitosis events missed by the prediction (false negatives),
- **BFP(i)**: number of incorrect mitosis predictions not supported by ground truth (false positives).

The F1-based score is defined as:

$$\mathrm{BC}(i) = \frac{2 \cdot \mathrm{BTP}(i)}{2 \cdot \mathrm{BTP}(i) + \mathrm{BFP}(i) + 2 \cdot \mathrm{BFN}(i)} \tag{14}$$

Cell Cycle Accuracy (CCA)[2]: CCA measures how well the predicted cell cycle length distribution aligns with the ground truth. The cell cycle is defined as the time span from a cell's birth (e.g., mitosis) to its termination (e.g., another division or disappearance). CCA is computed as one minus the maximal absolute difference between the cumulative distribution functions (CDFs) of predicted and ground truth cycle lengths:

$$\mathrm{CCA} = 1 - \max_t \left(|\mathrm{CDF}_{\mathrm{pr}}(t) - \mathrm{CDF}_{\mathrm{gt}}(t)| \right) \tag{15}$$

4 Supplementary Figure

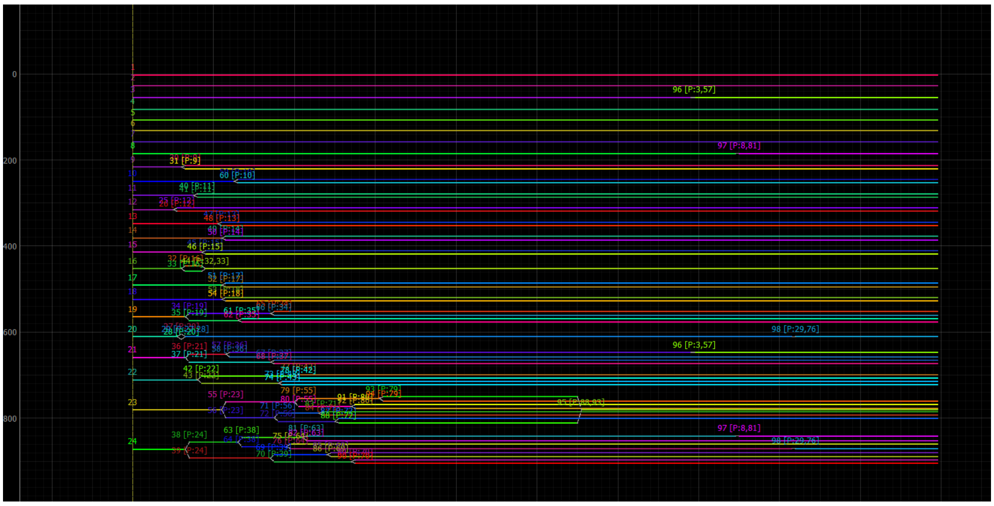

Fig. S2: **Graphical snapshot of lineage tree identifying 6 fusion events in one synthetic dataset.**

5 Supplementary Table

TABLE S4: Sensitivity analysis of ILP tracking by varying mitosis cost (w_m) and fusion cost (w_f) combinations across four datasets: HeLa and T98G cell lines with either ground truth or Detectron2-generated segmentation masks. Transition cost weight $w_t = 1$, appearance cost $w_a = 20$, disappearance cost $w_d = 20$, and maximum link distance $d_{max} = 50$ pixels.

CTC Metrics	Trackpy	Exp1	Exp2	Exp3	Exp4	Exp5	Exp6	Exp7	Exp8	Exp9*	Exp10	Exp11	Exp12	Exp13	Exp14	Exp15
w_m / w_f	n/a	(0, 0)	(1, 0)	(5, 0)	(10, 0)	(1, 10)	(5, 10)	(10, 10)	(1, 20)	(5, 20)	(7.5, 20)	(10, 20)	(15, 20)	(17.5, 20)	(18.5, 20)	(18.5, 25)
Hela CTC Ground Truth Mask																
TRA	0.981	0.983	0.983	0.983	0.983	0.983	0.983	0.983	**0.983**	0.983	0.983	0.983	0.982	0.982	0.982	0.982
CHOTA	0.811	0.871	0.871	0.871	0.861	0.871	0.871	0.861	**0.871**	0.871	0.861	0.861	0.791	0.780	0.780	0.780
BIO(0)	0.357	0.718	0.718	0.718	0.683	0.744	0.744	0.709	**0.744**	0.744	0.709	0.709	0.492	0.403	0.403	0.403
OP_CLB(0)	0.654	0.841	0.841	0.841	0.823	0.855	0.855	0.837	**0.855**	0.855	0.837	0.837	0.725	0.679	0.679	0.679
mitosis (9)	0	6	6	6	5	6	6	5	**6**	6	5	5	1	0	0	0
fusion (0)	0	2	2	2	2	0	0	0	0	0	0	0	0	0	0	0
Hela CTC Detectron2 Mask																
TRA	0.970	0.970	0.970	0.970	0.970	0.970	0.970	0.970	0.970	**0.970**	0.970	0.970	0.970	0.969	0.969	0.969
CHOTA	0.822	0.908	0.908	0.895	0.873	0.908	0.895	0.873	0.908	**0.895**	0.874	0.873	0.858	0.808	0.808	0.808
BIO(0)	0.363	0.607	0.607	0.622	0.582	0.608	0.623	0.583	0.613	**0.627**	0.572	0.588	0.542	0.438	0.438	0.438
OP_CLB(0)	0.657	0.781	0.781	0.788	0.767	0.782	0.789	0.767	0.785	**0.792**	0.763	0.770	0.746	0.693	0.693	0.693
mitosis (9)	0	10	10	9	5	10	9	5	10	**9**	6	5	4	1	1	1
fusion (0)	0	8	4	4	4	3	3	3	0	**0**	0	0	0	0	0	0
T98G Human Ground Truth Mask																
TRA	0.999	0.999	0.999	0.999	0.999	0.999	0.999	0.999	0.999	1.000	**1.000**	0.999	0.999	0.999	0.999	0.999
CHOTA	0.965	0.945	0.946	0.947	0.947	0.948	0.952	0.952	0.949	0.954	**0.953**	0.953	0.955	0.956	0.959	0.959
BIO(0)	0.555	0.780	0.797	0.890	0.865	0.822	0.908	0.883	0.820	0.903	**0.912**	0.887	0.863	0.791	0.698	0.698
OP_CLB(0)	0.772	0.885	0.893	0.942	0.929	0.907	0.951	0.939	0.907	0.950	**0.954**	0.941	0.929	0.892	0.845	0.845
mitosis (8)	0	14	13	8	7	12	8	7	13	9	**8**	7	6	4	2	2
fusion (0)	0	7	7	6	6	3	3	3	0	0	**0**	0	0	0	0	0
T98G Detectron2 Mask																
TRA	0.923	0.920	0.921	0.921	0.922	0.922	0.922	0.923	0.922	0.923	0.923	0.923	0.923	0.923	**0.923**	0.923
CHOTA	0.822	0.798	0.800	0.801	0.805	0.807	0.812	0.815	0.805	0.810	0.810	0.815	0.813	0.811	**0.811**	0.811
BIO(0)	0.319	0.305	0.308	0.322	0.354	0.322	0.346	0.377	0.325	0.347	0.359	0.380	0.431	0.390	**0.400**	0.400
OP_CLB(0)	0.595	0.577	0.579	0.588	0.607	0.591	0.605	0.623	0.594	0.607	0.614	0.626	0.652	0.631	**0.636**	0.636
mitosis (8)	0	61	59	45	28	59	44	28	60	44	37	28	16	10	**8**	8
fusion (0)	0	56	56	56	56	28	28	28	0	0	0	0	0	0	**0**	0

Trackpy: Trackpy baseline without ILP optimization; w_m: mitosis cost weight; w_f: fusion cost weight; *Exp9 represents the default configuration ($w_m = 5$, $w_f = 20$) used throughout this work. Best performance values are shown in bold. Mitosis and fusion rows show detected event counts (ground truth values in parentheses).

TABLE S5: CTC metrics of Fluo-N2DH-SIM+ data set

Metrics	GT	TS	ILP
TRA	1	0.9993	**0.9994**
SEG	1	1.0000	1.0000
DET	1	1.0000	1.0000
IDF1	1	0.9547	**0.9620**
MOTA	1	1.0000	1.0000
HOTA	1	0.9686	**0.9743**
CHOTA	1	0.9771	**0.9779**
LNK	1	0.9944	**0.9953**
CT	1	0.8156	**0.8508**
TF	1	1.0000	1.0000
CCA	1	1.0000	1.0000
MT	1	1.0000	1.0000
ML	0	0.0000	0.0000
IDSW	0	0	0
TP	2607	2607	2607
FN	0	0	0
FP	0	0	0
Precision	1	1.0000	1.0000
Recall	1	1.0000	1.0000
BIO	1	0.9182	**0.9327**
OP_{CSB}	1	0.9563	**0.9640**

GT: Ground truth; ILP: this work; TS: TRACKASTRA; Best performance values are shown in bold.

References

[1] M. Maška, V. Ulman, D. Svoboda, P. Matula, P. Matula, C. Ederra, A. Urbiola, T. España, S. Venkatesan, D. M. Balak, P. Karas, T. Bolcková, M. Štreitová, C. Carthel, S. Coraluppi, N. Harder, K. Rohr, K. E. G. Magnusson, J. Jaldén, H. M. Blau, O. Dzyubachyk, P. Křížek, G. M. Hagen, D. Pastor-Escuredo, D. Jimenez-Carretero, M. J. Ledesma-Carbayo, A. Muñoz Barrutia, E. Meijering, M. Kozubek, C. Ortiz-de Solórzano, A benchmark for comparison of cell tracking algorithms, Bioinformatics 30 (11) (2014) 1609–1617. doi:10.1093/bioinformatics/btu080.

[2] T. Kaiser, V. Ulman, B. Rosenhahn, Chota: A higher order accuracy metric for cell tracking, in: A. Del Bue, C. Canton, J. Pont-Tuset, T. Tommasi (Eds.), Computer Vision – ECCV 2024 Workshops, Springer Nature Switzerland, Cham, 2025, pp. 122–138.

[3] P. Dendorfer, A. Osep, A. Milan, K. Schindler, D. Cremers, I. Reid, S. Roth, L. Leal-Taixé, MOTChallenge: A Benchmark for Single-Camera Multiple Target Tracking, International Journal of Computer Vision 129 (4) (2021) 845–881. doi:10.1007/s11263-020-01393-0.

[4] B. Wu, R. Nevatia, Tracking of multiple, partially occluded humans based on static body part detection (2006). doi:10.1109/cvpr.2006.312.

[5] J. Luiten, A. Osep, P. Dendorfer, P. Torr, A. Geiger, L. Leal-Taixé, B. Leibe, Hota: A higher order metric for evaluating multi-object tracking, International Journal of Computer Vision 129 (2) (2021) 548–578. doi:10.1007/s11263-020-01375-2.
